# Mortality trends and demographic-geographic disparities of autoimmune liver diseases among U.S. adults aged ≥45 years, 1999-2023

**DOI:** 10.3389/fimmu.2026.1762095

**Published:** 2026-02-09

**Authors:** Chenjie Qiu, Honglei Shi, Liangliang Dai

**Affiliations:** 1 Department of General Surgery, Changzhou Hospital of Traditional Chinese Medicine, Changzhou, China; 2 Department of Urology, Wujin Hospital Affiliated With Jiangsu University, Changzhou, China; 3 Department of Urology, The Wujin Clinical College of Xuzhou Medical University, Changzhou, China

**Keywords:** autoimmune liver diseases, CDC WONDER, health disparities, mortality trends, United States

## Abstract

**Objective:**

To analyze the temporal mortality trends and demographic/geographic disparities of autoimmune liver diseases (ALDs) among individuals aged 45 years or older in the U.S. from 1999 to 2023, and provide evidence for targeted prevention and control strategies.

**Methods:**

Based on the CDC WONDER database, multiple-cause-mention ALD-related deaths (AIH, PBC, PSC) were identified using ICD-10 codes. Age-adjusted mortality rates (AAMRs), annual percent changes (APC/AAPC) were calculated, and trend analyses were conducted via Joinpoint regression. The study population was restricted to decedents aged ≥45 years due to data availability constraints.

**Results:**

Total multiple-cause-mention ALD-related deaths increased by 165.05%, with AAMR rising from 1.65 to 2.74 per 100,000 (AAPC = 2.48). Females had a higher AAMR (2.97 per 100,000) than males (2.53 per 100,000) in 2023. The 45–54 age group had the fastest AAPC (2.56), and non-Hispanic Black individuals had the highest AAPC (2.94). The West had the highest AAMR (3.24 per 100,000) in 2023. Based on data from 1999-2020, in 2020, AAMR in nonmetropolitan areas (3.09 per 100,000) surpassed that in metropolitan areas, with widening urban-rural gaps.

**Conclusion:**

U.S. ALD-related multiple-cause-mention mortality continues to rise among adults aged ≥45 years, with significant disparities across age, race/ethnicity, and urban-rural regions. Improving healthcare access for vulnerable populations and developing new therapies are essential to reduce the disease burden.

## Introduction

Autoimmune liver diseases (ALDs) comprise a group of chronic, progressive inflammatory disorders characterized by immune-mediated injury to hepatic tissue. The primary subtypes include autoimmune hepatitis (AIH), primary biliary cholangitis (PBC), and primary sclerosing cholangitis (PSC) (1). Although the prevalence of ALDs is lower than that of other major liver diseases (2), the clinical spectrum is broad, ranging from asymptomatic presentations to acute hepatitis, cirrhosis, and liver failure. Approximately one third of patients are diagnosed at the stage of cirrhosis, significantly increasing the risk of end-stage liver disease and contributing to disability and mortality among adults with chronic liver disease worldwide (3).

Although disruption of immune tolerance is the core pathogenic mechanism of ALDs, the subtypes differ substantially in target organs, immunological markers, and clinical phenotypes. AIH is characterized by diffuse hepatocellular inflammatory necrosis. PBC mainly involves immune-mediated injury to the epithelial cells of the small intrahepatic bile ducts. PSC affects both intrahepatic and extrahepatic bile ducts, leading to progressive biliary destruction, biliary cirrhosis, and ultimately portal hypertension (2, 4). These conditions are chronic and progressive, and treatment options remain limited with minimal change over the past two decades. At present, ursodeoxycholic acid (UDCA) is the only first-line therapy approved for PBC, and international guidelines recommend its use in all PBC patients (5). Although liver transplantation has inherent limitations, it has become a routine and effective intervention in the U.S (6).

Epidemiological patterns of ALDs vary substantially across countries. In France, the prevalence of PBC and AIH is significantly higher than that of PSC. The incidence of AIH has increased, whereas that of PBC and PSC has declined. From a geographical perspective, AIH clusters in the southwest of France and PBC clusters in the east, while PSC shows no clear spatial aggregation. Demographically, PSC patients are younger and have a balanced sex distribution, whereas AIH and PBC predominantly affect middle-aged and older women. PSC is associated with the highest mortality rate and a fourfold higher need for liver transplantation compared with other ALD subtypes (7). In Sweden, the mortality rate of PSC patients is four times higher than the general population, and the demand for liver transplantation is also the highest. Young adults between 18 and 50 years exhibit a relatively higher mortality risk, which differs from conventional expectations (8). In the U.S., epidemiological data from 2011 to 2017 show that although ALD-related hospitalizations have declined, the healthcare burden has intensified. Hospitalization costs have increased markedly and in-hospital mortality rose from 4.67 percent to 5.43 percent. ALD primarily affects adults aged 65 and older, women, and White individuals, with most hospitalizations occurring at large teaching hospitals in the southern region (2). These trends highlight the complexity of ALD management and the growing economic burden associated with these diseases.

Despite considerable progress in clinical research, systematic analyses of long-term mortality trends and population-level disparities in ALDs remain limited. To address this gap, the present study analyzes national multiple-cause-mention mortality data from the U.S. from 1999 to 2023 among adults aged ≥45 years to investigate temporal mortality patterns across ALDs, identify demographic and geographic disparities, and provide evidence to support the development of targeted prevention and control strategies by health authorities.

## Materials and methods

### Data source and case definition

Mortality data were obtained from the Centers for Disease Control and Prevention (CDC) Wide-ranging ONline Data for Epidemiologic Research (WONDER) database, which provides national death registration records from 1999 to 2023. Multiple-cause-mention ALD-related deaths were identified using the International Classification of Diseases, 10th Revision (ICD-10) codes: AIH (K75.4), PBC (K74.3), and PSC (K83.0), which is the standard code for PSC in mortality data and has been used in multiple studies (9–12). Death certificates include underlying and multiple causes of death, demographic information (sex, age, race/ethnicity), and geographic characteristics (state of residence, census region, and urban-rural classification). All data are publicly available, de-identified, and exempt from requirements for individual informed consent.

Eligible death records met the following criteria: (a) AIH, PBC, or PSC was explicitly listed as one of the multiple causes of death (not necessarily the underlying cause), and (b) the decedent was aged ≥45 years. The study was restricted to individuals aged ≥45 years because the number of ALD-related deaths among those younger than 45 was below the CDC’s publication threshold (<10 deaths per year) in most years, precluding stable analysis. This age group constitutes the vast majority of ALD-related mortality.

Four sets of analytic variables were defined according to CDC and U.S. Census Bureau classification standards, including sex (male and female), age (45-54, 55-64, 65-74, 75-84, and ≥85 years), race/ethnicity (Hispanic, NH Black, NH White, and NH Other races (a composite group including, for example, Asian, Native Hawaiian, Pacific Islander, American Indian, and Alaska Native individuals)), and geographic region and urbanization status, classified into four census regions (Northeast, Midwest, South, and West) and two urbanization levels (metropolitan areas with populations ≥50,000 and nonmetropolitan areas with populations <50,000) (13).

### Indicator definitions

The crude death rate (CDR) is defined as the number of ALD-related deaths in a given year or subgroup divided by the corresponding population, multiplied by 100,000 (per 100,000 population). The age-adjusted mortality rate (AAMR) is calculated using direct standardization to the 2000 U.S. standard population to account for differences in age structure. 95% confidence intervals (CIs) for AAMRs were estimated using the normal approximation method, which is the standard approach for national mortality data in the CDC WONDER database. We note that for subgroups with very few deaths, the accuracy of this method may be reduced. The annual percent change (APC) quantifies the average annual rate of change in AAMR within each identified trend segment. The average annual percent change (AAPC) reflects the overall direction and magnitude of change across the entire study period.

### Statistical analysis

Descriptive statistics were used to summarize the number and percentage of ALD-related deaths and calculate CDRs or AAMRs for the total population and all subgroups. Temporal trends in AAMR were assessed using Joinpoint regression analysis, which identifies statistically significant inflection points and partitions the time series into distinct trend segments. The Joinpoint regression analysis was conducted using the Joinpoint Regression Program. The analysis adopted a log-linear model, with the error option set to standard error (homogeneous variance). The maximum number of joinpoints allowed in the model was set to 4, and model selection was based on permutation tests (4,499 permutations were performed). The overall significance level was set at 0.05. The annual percentage change (APC) for each trend segment and the average annual percentage change (AAPC) for the entire study period, along with their 95% confidence intervals, were all calculated using the parametric method. AAPCs and 95% CIs were computed for each demographic and geographic subgroup. All analyses were conducted using R software and the Joinpoint Regression Program. Subtype-specific analyses for AIH, PBC, and PSC were also performed.

### Ethical statement

This study analyzed publicly available, de-identified data from the CDC and the U.S. Census Bureau. The use of these data is consistent with ethical standards for public health surveillance research and does not require institutional review board approval or informed consent.

## Results

### Overall mortality trend

Between 1999 and 2023, total multiple-cause-mention deaths attributable to ALDs among individuals aged ≥45 years increased from 1,568 to 4,156, representing a 165.05% rise. The AAMR likewise increased from 1.65 per 100,000 population in 1999 (95% CI: 1.57-1.73) to 2.74 per 100,000 in 2023 (95% CI: 2.66-2.83), with an AAPC of 2.48 (95% CI: 1.64-3.33). All differences were statistically significant, indicating a sustained increase in the overall disease burden of ALDs (**Table 1**; **Supplementary Table S1**).

**Table 1:** Age-adjusted mortality rates (AAMR) and annual percent changes.

Characteristic	Death			AAMR		
	1999	2023	Percent change (%)	1999 (95% CI)	2023 (95% CI)	AAPC (95% CI)
Overall	1568	4156	165.05	1.65 (1.57 to 1.73)	2.74 (2.66 to 2.83)	2.48 (1.64 to 3.33)*
Sex						
Female	972	2447	151.75	1.70 (1.59 to 1.81)	2.97 (2.85 to 3.09)	2.64 (1.70 to 3.59)*
Male	596	1709	186.74	1.56 (1.43 to 1.69)	2.53 (2.41 to 2.65)	2.08 (1.84 to 2.32)*
Age#						
45–54 years	120	248	106.67	0.33 (0.27 to 0.39)	0.61 (0.54 to 0.69)	2.56 (2.06 to 3.06)*
55–64 years	231	617	167.10	0.97 (0.85 to 1.10)	1.47 (1.36 to 1.59)	1.64 (1.15 to 2.13)*
65–74 years	400	1147	186.75	2.17 (1.96 to 2.38)	3.31 (3.12 to 3.50)	2.17 (1.00 to 3.36)*
75–84 years	512	1311	156.05	4.19 (3.83 to 4.55)	7.14 (6.75 to 7.52)	2.39 (1.76 to 3.03)*
85+ years	305	833	173.11	7.34 (6.52 to 8.17)	13.45 (12.53 to 14.36)	2.14 (1.83 to 2.46)*
Census Region						
Northeast	336	730	117.26	1.66 (1.48 to 1.84)	2.61 (2.42 to 2.80)	1.96 (0.89 to 3.04)*
Midwest	361	959	165.65	1.62 (1.45 to 1.79)	2.97 (2.78 to 3.16)	2.48 (2.16 to 2.80)*
South	497	1358	173.24	1.50 (1.37 to 1.64)	2.35 (2.23 to 2.48)	2.40 (1.33 to 3.47)*
West	374	1109	196.52	1.98 (1.77 to 2.18)	3.24 (3.05 to 3.43)	2.19 (0.99 to 3.41)*
Race						
Hispanic	123	477	287.80	2.66 (2.17 to 3.15)	2.99 (2.71 to 3.26)	0.81 (0.31 to 1.31)*
NH Black	105	428	307.62	1.32 (1.06 to 1.57)	2.84 (2.56 to 3.12)	2.94 (2.44 to 3.44)*
NH White	1290	2936	127.60	1.62 (1.54 to 1.71)	2.66 (2.56 to 2.75)	2.43 (1.59 to 3.27)*
NH Other	45	306	580.00	1.76 (1.25 to 2.40)	2.91 (2.58 to 3.24)	1.78 (0.01 to 3.58)*
Urbanization##						
Metropolitan	1277	3447	169.93	1.63 (1.54 to 1.72)	2.63 (2.54 to 2.72)	2.29 (1.53 to 3.06)*
Nonmetropolitan	291	709	143.64	1.61 (1.43 to 1.80)	3.09 (2.86 to 3.31)	2.97 (1.55 to 4.41)*

The AAPC was calculated for the period 1999–2023. #Age groups used crude mortality rates for calculation. ##Urban-rural analysis (AAMR and AAPC) is for the period 1999-2020. The table lists the death numbers for 2023, but due to the lack of urban-rural classification data after 2020, the corresponding 2023 AAMR has not been calculated. The AAMR values shown for urbanization are for the year 2020. *Means statistical significance. (APC/AAPC) for autoimmune liver diseases in the United States, 1999-2023.

### Sex differences

**Figure 1:**
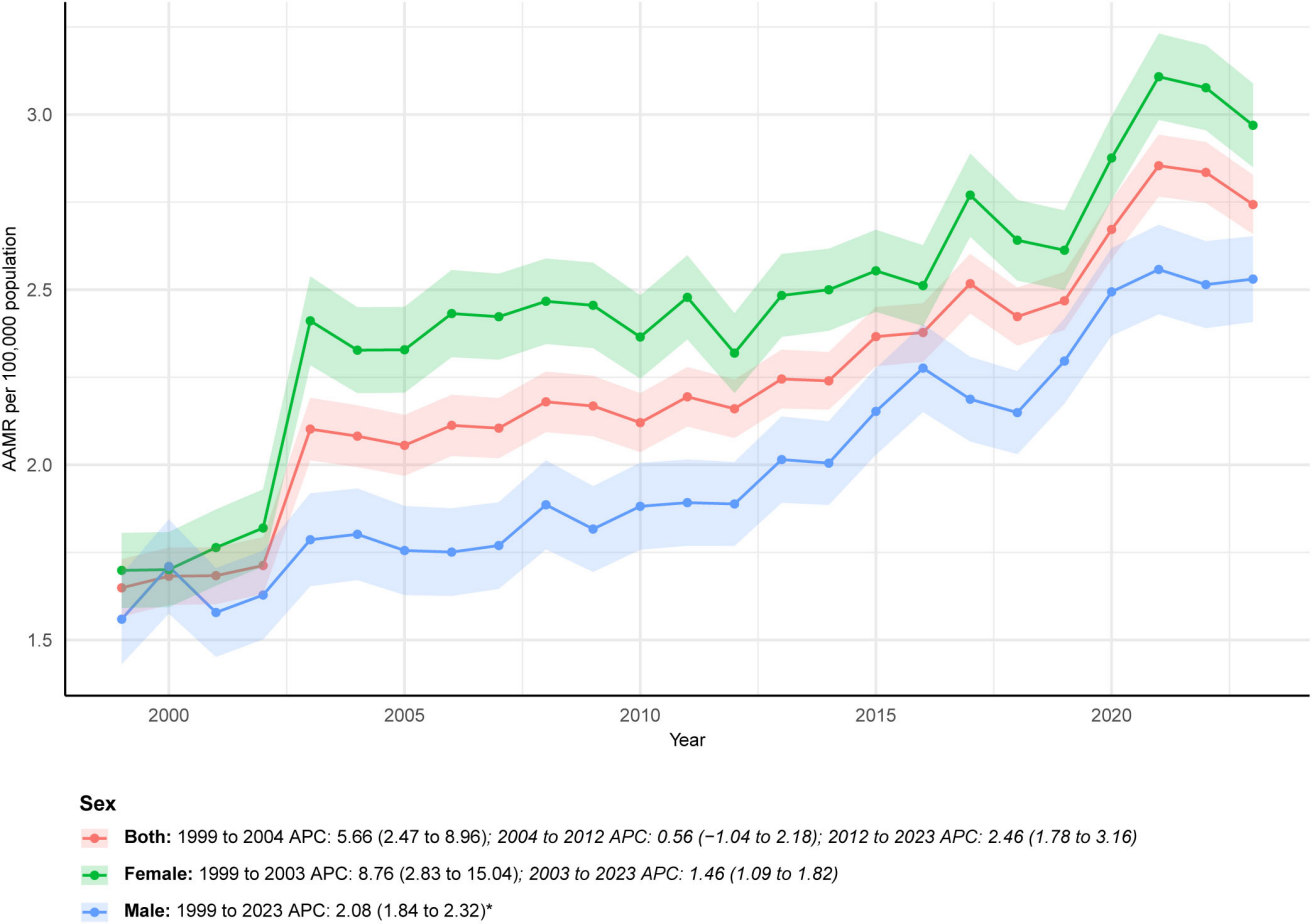
ALDs AAMR by sex (1999-2023).

Deaths among males increased by 186.74%, rising from 596 in 1999 to 1,709 in 2023, which exceeded the 151.75% increase among females (from 972 to 2,447). However, the AAMR in 2023 was higher among females (2.97 per 100,000, 95% CI: 2.85-3.09) than among males (2.53 per 100,000, 95% CI: 2.41-2.65). Over time, the AAPC among males remained stable, with an APC of 2.08 (95% CI: 1.84-2.32) between 1999 and 2023. In contrast, the female trend exhibited marked fluctuations, with a rapid increase from 1999 to 2003 (APC: 8.76, 95% CI: 2.83-15.04) followed by a slower increase thereafter (APC: 1.46, 95% CI: 1.09-1.82) (**Figure 1**; **Table 1**; **Supplementary Tables S2**, **S3**).

### Age differences

**Figure 2:**
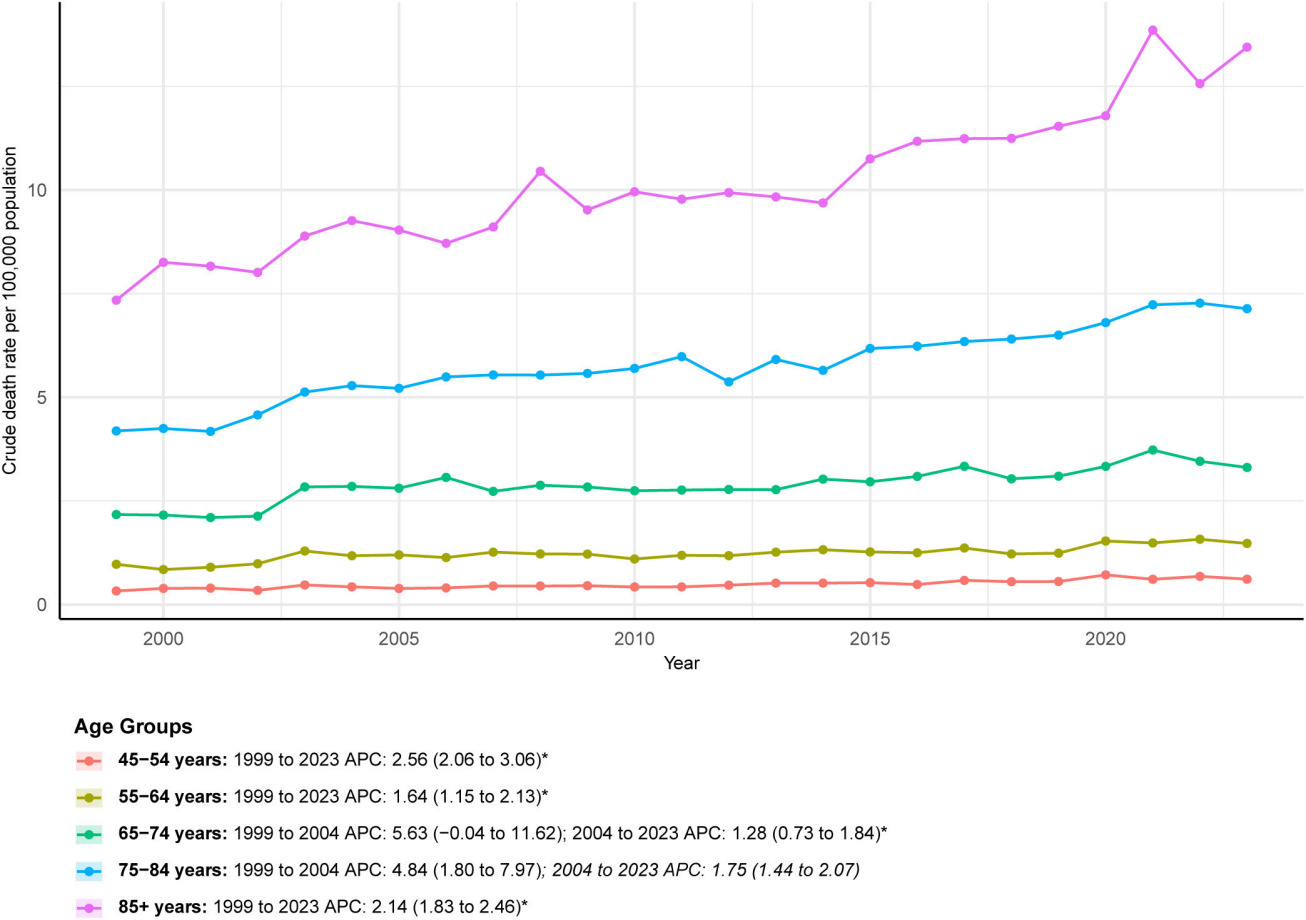
ALDs Crude death rate by age groups (1999-2023).

CDRs increased sharply with age. In 2023, adults aged 85 years or older had the highest CDR at 13.45 per 100,000 (95% CI: 12.53-14.36), which was more than 22 times the rate among adults aged 45–54 years (0.61 per 100,000, 95% CI: 0.54-0.69). The fastest increase in AAPC occurred in the 45–54 age group (2.56, 95% CI: 2.06-3.06). The 65–74 age group experienced the largest rise in the number of deaths, increasing by 186.75% (from 400 to 1,147). Notably, growth in the 65–74 and 75–84 age groups slowed considerably after 2004, with APCs decreasing from 5.63 and 4.84 in 1999–2004 to 1.28 and 1.75 in 2004-2023, respectively (**Figure 2**; **Table 1**; **Supplementary Table S2**).

### Racial/ethnic differences

**Figure 3:**
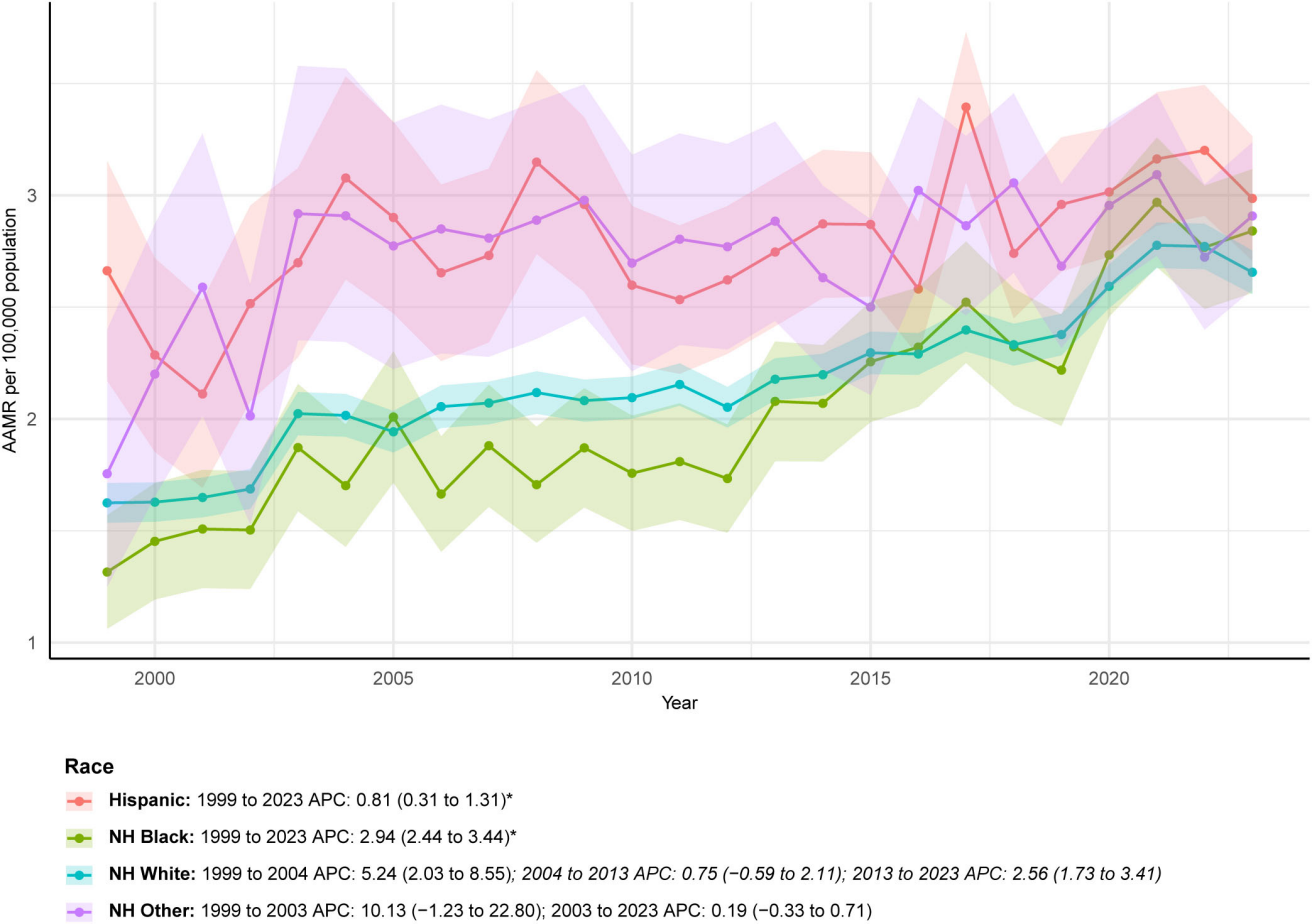
ALDs AAMR by race/ethnicity (1999-2023).

Across racial/ethnic groups, the NH Other population experienced the largest relative increase in deaths (580.00%), with deaths rising from 45 to 306, although their AAPC was modest (1.78, 95% CI: 0.01-3.58). This category encompasses heterogeneous groups, and the large percentage increase should be interpreted with caution due to potential differences in risk profiles, coding, and access to care among the constituent populations. The NH Black population exhibited the fastest AAPC at 2.94 (95% CI: 2.44-3.44), and the AAMR in this group increased from 1.32 per 100,000 in 1999 (95% CI: 1.06-1.57) to 2.84 per 100,000 in 2023 (95% CI: 2.56-3.12). By contrast, the Hispanic population had the lowest AAPC (0.81, 95% CI: 0.31-1.31), and their AAMR increased only slightly from 2.66 per 100,000 in 1999 (95% CI: 2.17-3.15) to 2.99 per 100,000 in 2023 (95% CI: 2.71-3.26) (**Figure 3**; **Table 1**; **Supplementary Tables S2**, **S4**).

### Regional differences

**Figure 4:**
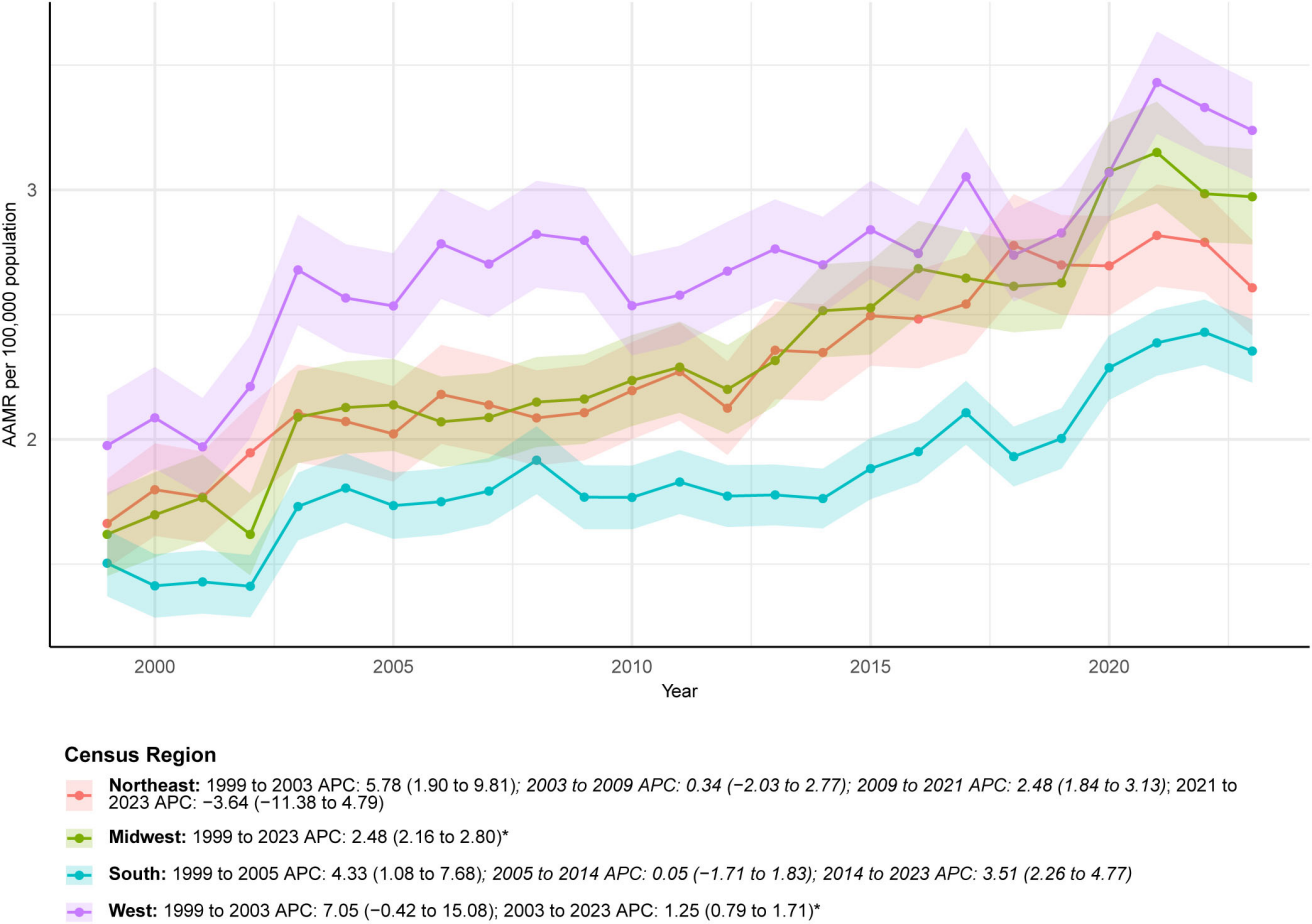
ALDs AAMR by U.S. census regions (1999-2023).

Regionally, the West experienced the largest increase in deaths (196.52%), with counts rising from 374 in 1999 to 1,109 in 2023. In 2023, the West also had the highest AAMR at 3.24 per 100,000 (95% CI: 3.05-3.43). The Midwest showed the most stable long-term pattern, with an AAPC of 2.48 (95% CI: 2.16-2.80) and no significant fluctuations. The Northeast experienced a brief decline from 2021 to 2023 (APC: -3.64, 95% CI: -11.38-4.79), though this change was not statistically significant (**Figure 4**; **Table 1**; **Supplementary Tables S2**, **S5**).

### Urban-rural differences

**Figure 5:**
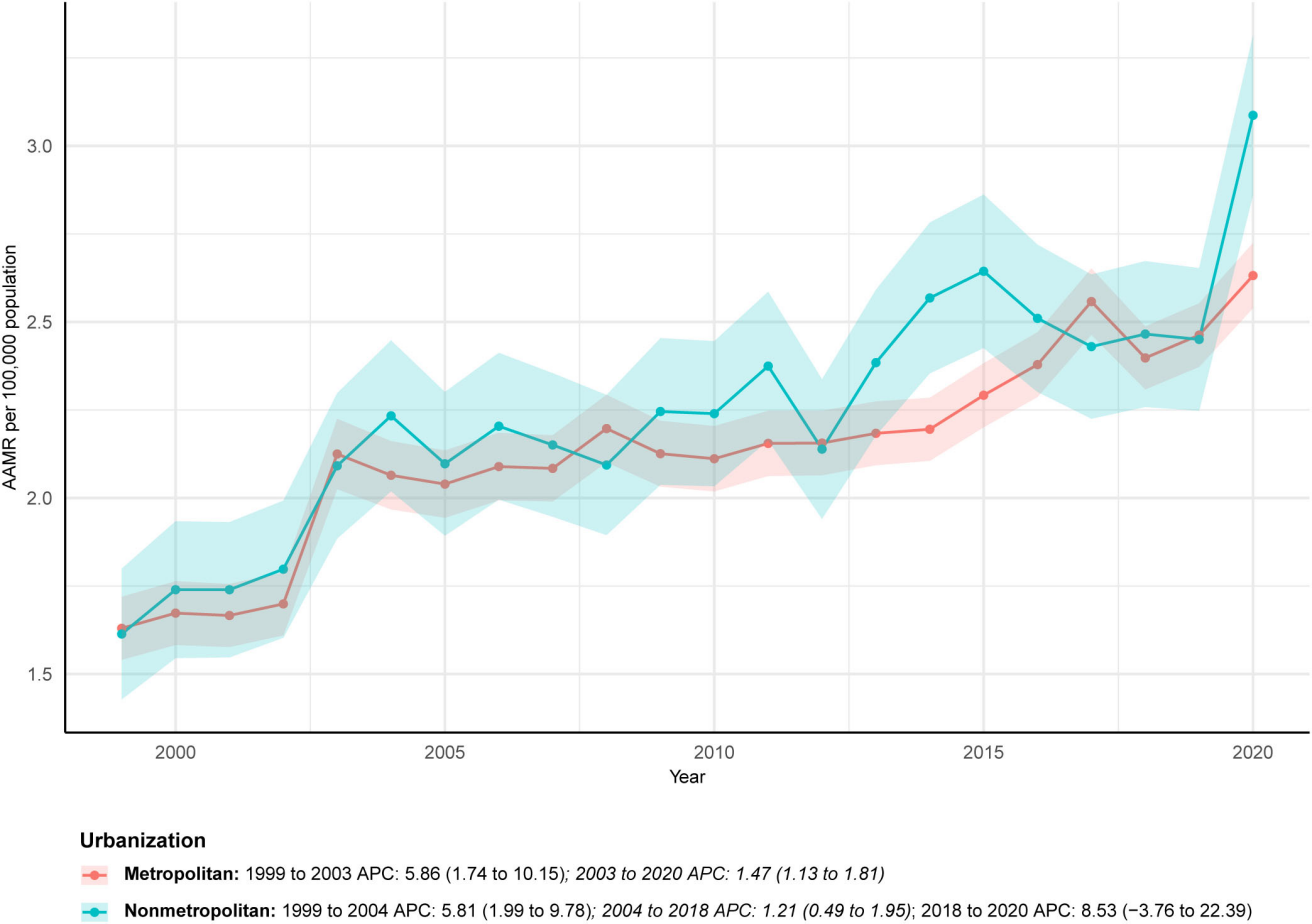
ALDs AAMR by urban-rural level (1999-2020).

The urban-rural difference analysis is based on data from 1999 to 2020, as the source database does not provide urban-rural classification data after 2020. In 2020, the AAMR of ALDs in nonmetropolitan areas (3.09 per 100,000, 95% CI: 2.86-3.31) surpassed that in metropolitan areas (2.63 per 100,000, 95% CI: 2.54-2.72). Nonmetropolitan areas also showed a short-term surge between 2018 and 2020, with an APC of 8.53 (95% CI: -3.76-22.39). Over the long term, the AAPC was higher in nonmetropolitan populations (2.97, 95% CI: 1.55-4.41) than in metropolitan populations (2.29, 95% CI: 1.53-3.06), suggesting widening urban-rural disparities (**Figure 5**; **Table 1**; **Supplementary Tables S2**, **S6**).

### Subtype-specific analysis

From 2003 to 2023, deaths related to AIH increased from 435 to 841, representing a rise of 93.33%. During the same period, the AAMR increased from 0.42 to 0.56 per 100,000 population, with an AAPC of 1.77 (95% CI: 1.19 to 2.35), indicating a significant upward trend (**Supplementary Figure S1**; **Supplementary Table S7**). The increase in deaths among males was substantially higher than among females (164.38% vs 79.01%), and the AAPC was also higher in males (3.14 vs 1.25). The largest increase in deaths occurred among individuals aged 85 years and older (361.90%), with an AAPC of 4.27, whereas no significant change in AAMR was observed in the 65–74 group. All four census regions experienced significant increases in AAMR, with the South showing the largest rise (111.41%). AAMR increased significantly in metropolitan areas (AAPC 1.71), while no significant trend was observed in nonmetropolitan areas. Deaths among Hispanic showed the largest increase (173.33%), and the AAPC among NH Black (2.37) exceeded that of NH White (1.65).

From 1999 to 2023, deaths related to PBC declined slightly from 509 to 503, corresponding to a decrease of 1.18%. Over the same period, the AAMR declined from 0.51 to 0.35 per 100,000 population, with an AAPC of -1.56 (95% CI: -1.88 to -1.23), demonstrating a significant downward trend (**Supplementary Figure S2**; **Supplementary Table S8**). Female deaths decreased by 7.85%, accompanied by a significant decline in AAMR (AAPC-1.74). In contrast, male deaths increased by 46.03%, although no significant change in AAMR was observed. Both the number of deaths and AAMR declined significantly among individuals aged 45–74 years. For example, deaths among those aged 45 to 54 years decreased by 47.50%, with an AAPC of -1.34. Conversely, deaths among individuals aged 85 years and older increased by 134.29%, with an AAPC of 1.26. AAMR declined significantly in all four census regions, with the largest decrease observed in the West (AAPC -3.31). Significant declines in AAMR were observed in both metropolitan and nonmetropolitan areas. Deaths among NH White decreased by 11.24%, whereas deaths among Hispanic increased by 39.53%, despite a significant decline in AAMR (AAPC -2.36).

From 1999 to 2023, deaths related to PSC increased from 1,059 to 2,857, representing a rise of 169.78%. The AAMR increased from 1.09 to 1.91 per 100,000 population, with an AAPC of 2.24 (95% CI: 1.89 to 2.59), indicating a significant upward trend (**Supplementary Figure S3**; **Supplementary Table S9**). Deaths among both females and males more than doubled, increasing by 170.91% and 168.67%, respectively. The AAPC was higher in females (2.71) than in males (1.63). The largest increase in deaths occurred among individuals aged 65 to 74 years (229.69%), with an AAPC of 2.69. Although deaths among those aged 45 to 54 years increased by 92.50%, no significant change in AAMR was observed in this group. AAMR increased significantly across all four census regions, with particularly large increases in the Midwest and West, where deaths rose by 201.36% and 205.18%, respectively. AAMR increased significantly in metropolitan areas (AAPC 1.99), while no significant trend was detected in nonmetropolitan areas. The largest increase in deaths occurred among NH Other races (502.70%). AAMR increased significantly among NH Black and NH White, whereas no significant change was observed among Hispanic.

## Discussion

This study, based on 25 years of national multiple-cause-mention mortality data from the U.S. among individuals aged ≥45 years, reveals a persistent and concerning rise in mortality associated with ALDs and identifies notable demographic and geographic disparities. These findings not only confirm the increasing burden of ALDs but also delineate high-risk population groups, offering valuable evidence to inform clinical practice and guide public health policy development.

The AAPC in the overall AAMR for ALDs was 2.48, and the total number of multiple-cause-mention deaths increased by 165.05% over the study period. This sustained rise contrasts sharply with the reported decline in hospitalization rates for ALDs in the U.S. between 2011 and 2017, yet aligns with evidence demonstrating increased healthcare burden during the same period, including rising hospitalization costs and in-hospital mortality (2). Several interrelated factors may contribute to this upward mortality trend. First, advances in diagnostic technologies, particularly the broader application of autoantibody testing and imaging, may have reduced underdiagnosis, especially in asymptomatic or early-stage cases that were previously overlooked. The diagnosis of AIH commonly relies on a spectrum of autoantibodies, including antinuclear antibodies (ANA), smooth muscle antibodies (SMA), liver-kidney microsomal (LKM) antibodies, and soluble liver antigen (SLA) antibodies (14). In PBC, the M2 subtype of antimitochondrial antibody (AMA M2) serves as a highly specific biomarker for current or future disease and is detectable in approximately 90-95% of patients (15). Although multiple autoantibodies may be present in PSC, none are disease specific; the most extensively studied is antineutrophil cytoplasmic antibody (ANCA), which appears in 65-95% of cases (16). Diagnosis of PSC typically relies on magnetic resonance imaging (MRI) to identify biliary cholestasis or strictures (17). Second, therapeutic progress for ALDs remains limited. Since the 1970s, treatment for AIH has largely depended on corticosteroids such as prednisone and the immunosuppressant azathioprine, which prevent further liver injury in about 80% of patients but are associated with substantial adverse effects (17). Ursodeoxycholic acid (UDCA) is the only first-line therapy for PBC. However, up to 40% of patients exhibit an inadequate response. No effective medical therapy currently improves transplant-free survival in PSC, for which liver transplantation remains the only curative option. Recurrence occurs in 20-25% of patients within 5–10 years after transplantation (18). The temporal pattern of female mortality (a rapid rise before 2003 followed by a slower increase) coincides with the widespread adoption of UDCA, suggesting a potential mitigating effect of this therapy on disease progression in women (19). Finally, population aging in the U.S. is likely contributing to rising mortality. Because ALD-related mortality increases markedly with age, and the AAMR among adults aged ≥85 years is approximately 22-fold higher than that among adults aged 45–54 years, the growing proportion of older adults has amplified the overall mortality burden.

The sex-specific patterns identified in this study provide new insight into the well-recognized female predominance of ALDs. Although the AAMR in 2023 remained higher in women (2.97 per 100,000) than in men (2.53 per 100,000), the relative increase in mortality was greater in men (186.74%) than in women (151.75%). The long-term AAPC in men was stable at 2.08, whereas women exhibited a biphasic trajectory, with a rapid rise from 1999 to 2003 (APC: 8.76), followed by a slower increase thereafter (APC: 1.46). The early surge among women may be partially attributable to the clinical adoption of UDCA. After the U.S. FDA approved UDCA as the first-line therapy for PBC in 1997, it became a major therapeutic advance (20). Widespread use of UDCA likely mitigated disease progression in subsequent years. The 10-year survival rate among treated patients approaches 80%, whereas untreated PBC patients have an average survival of only 9–10 years, with approximately 25% developing liver failure during that period (21). Despite women having higher overall mortality, men exhibit distinct prognostic features. Male patients with AIH generally experience better long-term outcomes than females (22). In PBC, men tend to present with milder symptoms, whereas women more commonly experience pruritus, potentially leading to delayed diagnosis due to hormone-related differences (23). However, men have a higher likelihood of inadequate response to standard UDCA therapy and may face a substantially increased risk of hepatocellular carcinoma compared with women with PBC (21). The steady rise in male mortality may reflect underdiagnosis and greater disease severity at presentation, including more advanced fibrosis, a higher burden of complications, and poorer therapeutic response. These factors collectively may elevate mortality risk among men with ALDs.

Age-related differences reveal a shift in the distribution of risk. Although adults aged ≥85 years have the highest CDR, the 45-54-year age group exhibited the most rapid increase in mortality, with an AAPC of 2.56, and individuals aged 65–74 years experienced the largest absolute rise in deaths (186.75%). These findings diverge from the traditional expectation that increases in mortality are concentrated primarily among older adults, suggesting that middle-aged populations are encountering emerging vulnerabilities. Contributing factors may include rising prevalence of modifiable risk factors in this age group, such as obesity, metabolic syndrome, and environmental exposures, which may potentiate immune-mediated liver injury (24, 25). After 2004, the rate of increase in mortality among individuals aged 65–74 and 75–84 years slowed, further supporting the potential impact of improved clinical management. This stabilization may reflect earlier and broader adoption of UDCA therapy as well as more comprehensive monitoring and follow-up in older patients (21).

Racial and ethnic disparities highlight the influence of structural health inequities on ALD outcomes. NH Other individuals experienced the largest relative increase in deaths (580.00%), rising from 45 to 306 cases, although their long-term AAPC was comparatively modest (1.78). As noted, the “NH Other” category aggregates diverse racial groups, and the substantial percentage change should be interpreted with caution due to potential heterogeneity in baseline risks, access to care, and coding practices within this composite group. NH Black individuals exhibited the fastest AAPC growth (2.94), with AAMR increasing from 1.32 per 100,000 in 1999 to 2.84 per 100,000 in 2023. In contrast, Hispanics had the lowest AAPC (0.81), and their AAMR increased only slightly, from 2.66 to 2.99 per 100,000. Patterns in healthcare utilization further illustrate these disparities. Previous study found that NH White patients constituted the majority of PBC hospitalizations, increasing from 57.8% in 2007 to 71.2% in 2014. In comparison, NH Black and Hispanic patients accounted for only 4.1-6.3% and 8.6-10.9% of hospitalizations, respectively, and exhibited higher in-hospital mortality rates (4.3% for NH Black vs. 3.4% for NH White) (26). These findings align with the broader landscape of racial inequities in chronic liver disease care in the U.S., including delays in diagnosis among NH Black individuals, reduced access to hepatology specialists, and differences in treatment adherence (26–28). Structural disparities are also reflected in end-stage disease outcomes. Cholankeril et al. reported shifts in the racial/ethnic composition of liver transplant waiting lists for ALD-related end-stage liver disease in 2018. From 2002 to 2014, the proportion of NH White candidates declined markedly, while the number of Hispanic candidates remained stable. Importantly, both Hispanic and NH Black patients experienced higher waiting list mortality compared with NH White individuals, indicating a greater likelihood of dying before transplantation (29).

Geographic and urban-rural disparities further complicate the management of ALDs. The western U.S. experienced the largest relative increase in deaths (199.52%), rising from 374 to 1,109, and recorded the highest AAMR in 2023 (3.24 per 100,000). This pattern aligns with previous evidence showing higher in-hospital mortality among PBC patients in the western region compared with other parts of the country (26). Urban-rural differences were also notable. Based on data through 2020, in 2020, the AAMR in nonmetropolitan areas (3.09 per 100,000) surpassed that in metropolitan areas (2.63 per 100,000), and nonmetropolitan regions experienced a short-term surge in mortality between 2018 and 2020 (APC: 8.53). Over the long term, the AAPC remained higher in nonmetropolitan areas (2.97) than in metropolitan areas (2.29), indicating a widening urban-rural gap. The elevated mortality observed in the western region may reflect state-level variations in public health infrastructure, healthcare accessibility, and socioeconomic conditions (30). Similarly, disparities between urban and rural areas likely stem from uneven distribution of medical resources, higher levels of poverty, and greater comorbidity burden. Delayed specialist evaluation, limited availability of trained providers, and insufficiently equipped medical facilities in rural communities may further worsen ALD outcomes and contribute to higher mortality (31, 32).

This study has several limitations. First, mortality data are based on multiple-cause-of-death coding using ICD-10, which may introduce misclassification or underreporting, particularly when ALDs coexist with other chronic liver diseases. Furthermore, the use of multiple-cause-of-death data, while capturing a broader disease burden, may be influenced by changes in coding practices and clinical awareness over time. Second, due to the general limitations of ICD-10 coding for rare liver diseases, deaths involving other types of cholangitis may be assigned to K83.0, which may overestimate the number of PSC-related deaths. However, our study limited the analysis to deaths where PSC was explicitly listed as one of the “multiple causes of death” (rather than just as the underlying cause), which reduces the risk of false coding associations. Third, due to the privacy suppression rules of the U.S., mortality counts fewer than 10 are not reported, which necessitated restricting the analysis to individuals aged ≥45 years. Consequently, the findings are not generalizable to the entire U.S. population, and trends in younger age groups could not be assessed. Fourth, the race/ethnicity category “NH Other” combines heterogeneous populations, limiting the interpretability of trends within this group. Fifth, the study lacks individual-level information, thereby limiting the ability to explore mechanisms underlying the observed disparities. Finally, the ecological study design precludes causal inference. Trends may be influenced by unmeasured confounders, including shifts in population health behaviors or changes in healthcare policy.

Despite these limitations, the findings of this study have important implications for public health and clinical practice. To address the rising burden of ALDs, targeted strategies should focus on improving access to specialized hepatology care in nonmetropolitan and disadvantaged racial and ethnic communities, including reducing geographic barriers through expanded telemedicine, enhancing primary care physicians’ awareness of ALD symptoms and risk factors to minimize diagnostic delays, increasing research investment to develop new therapies for ALD subtypes with limited treatment options such as PSC, and strengthening surveillance of demographic and geographic trends to enable timely adjustments to prevention and management strategies. Furthermore, addressing broader social determinants of health, including poverty, healthcare accessibility, and environmental exposures, is essential for reducing persistent disparities in ALD-related mortality.

## Conclusion

This 25-year analysis of U.S. multiple-cause-mention mortality data among adults aged ≥45 years demonstrates a steady rise in ALD-related deaths, accompanied by widening disparities across age, race/ethnicity, and urban-rural populations. These patterns reflect not only improved diagnostic recognition but also persistent gaps in treatment effectiveness and healthcare access. In the context of the growing global burden of chronic immune-mediated diseases, the findings underscore the urgent need for equitable, patient-centered strategies aimed at slowing disease progression, reducing mortality, and improving outcomes for all individuals affected by ALDs.

## Data Availability

The original contributions presented in the study are included in the article/**Supplementary Material**. Further inquiries can be directed to the corresponding authors.

## Supplementary material

Supplementary Figure 1 **(A)** AIH AAMR by sex (1999-2023). **(B)** AIH crude death rate by age groups (1999-2023). **(C)** AIH AAMR by U.S. census regions (1999-2023). **(D)** AIH AAMR by urban-rural level (1999-2020).

Supplementary Figure 2 **(A)** PBC AAMR by sex (1999-2023). **(B)** PBC crude death rate by age groups (1999-2023). **(C)** PBC AAMR by U.S. census regions (1999-2023). **(D)** PBC AAMR by urban-rural level (1999-2020).

Supplementary Figure 3 **(A)** PSC AAMR by sex (1999-2023). **(B)** PSC crude death rate by age groups (1999-2023). **(C)** PSC AAMR by race/ethnicity (1999-2023). **(D)** PSC AAMR by U.S. census regions (1999-2023). **(E)** PSC AAMR by urban-rural level (1999-2020).
