# Supplementary figures and images for: Mortality trends and demographic-geographic disparities of autoimmune liver diseases among U.S. adults aged ≥45 years, 1999-2023
Source: Front Immunol. 2026 Feb 9;17:1762095. doi: 10.3389/fimmu.2026.1762095 (PMC12926130; doi:10.3389/fimmu.2026.1762095)

A

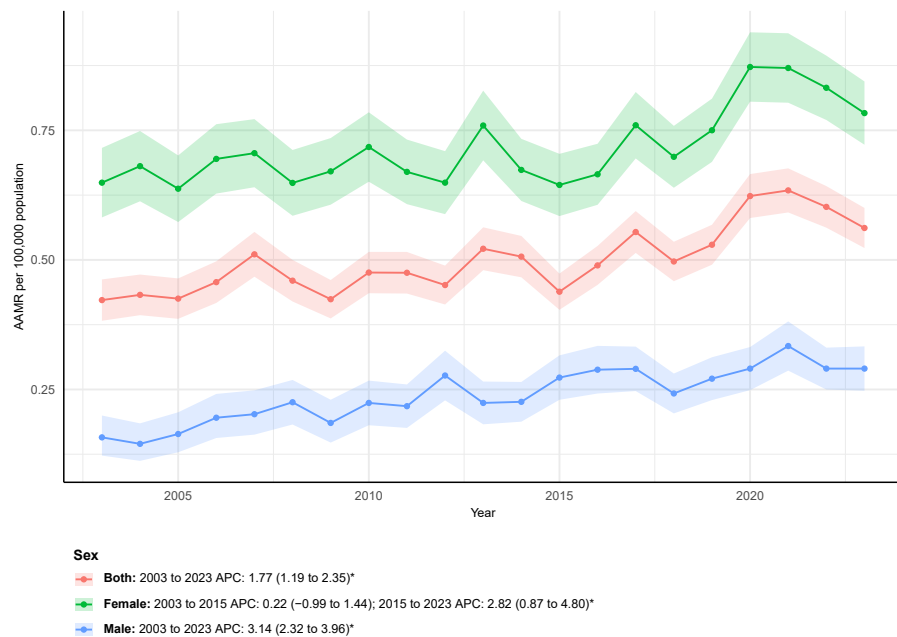

B

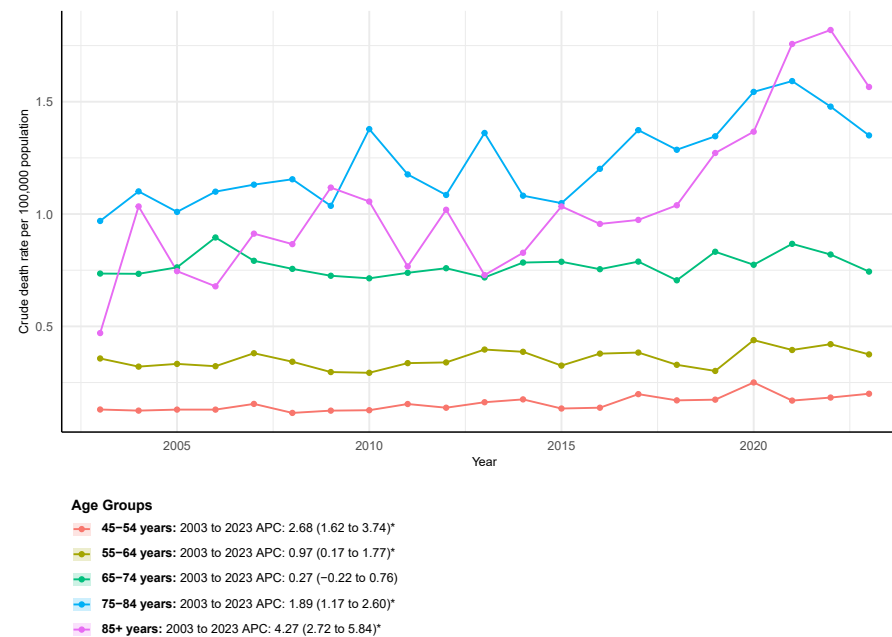

C

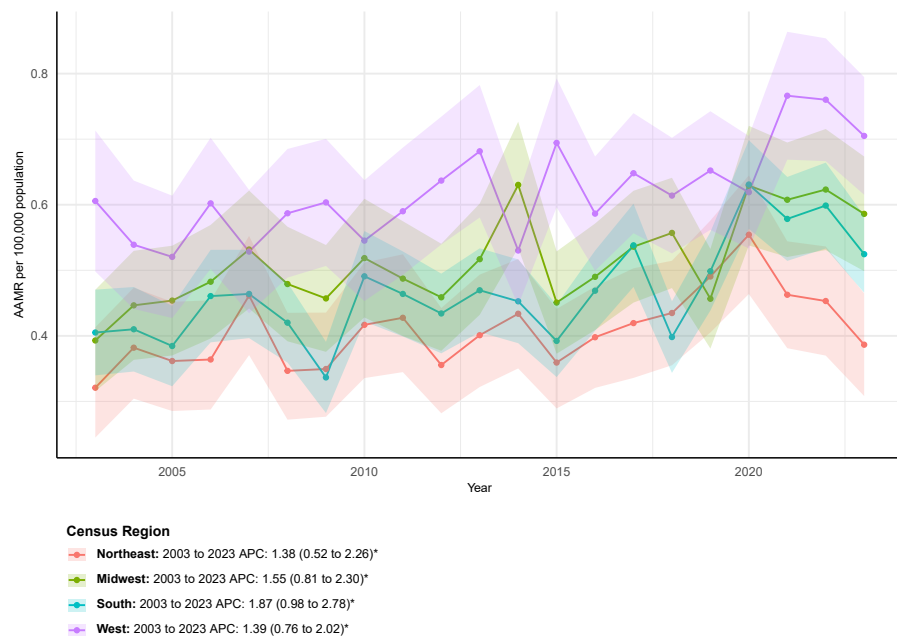

D

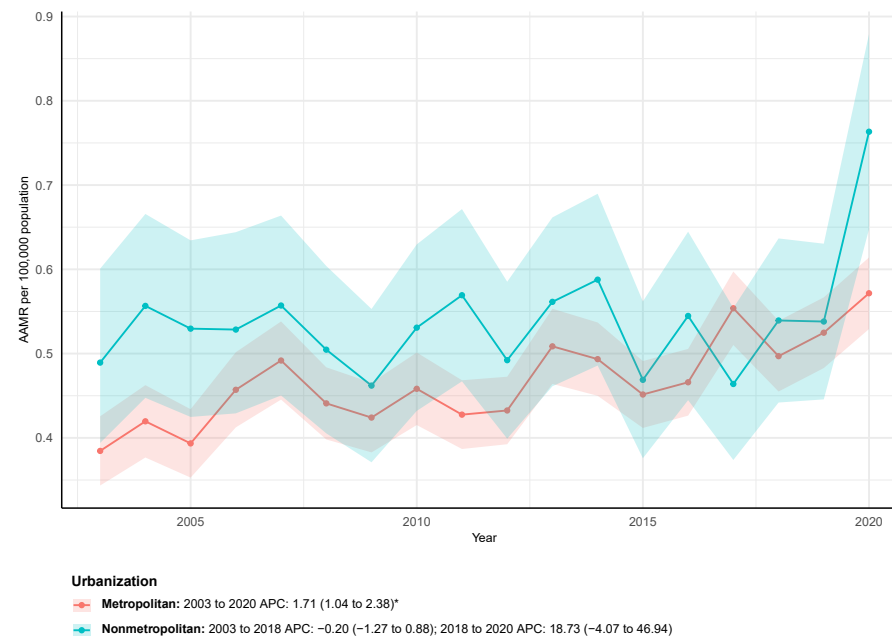

Supplement: Supplementary Figure 1 — (A) AIH AAMR by sex (1999-2023). (B) AIH crude death rate by age groups (1999-2023). (C) AIH AAMR by U.S. census regions (1999-2023). (D) AIH AAMR by urban-rural level (1999-2020). [file DataSheet1.pdf]

A

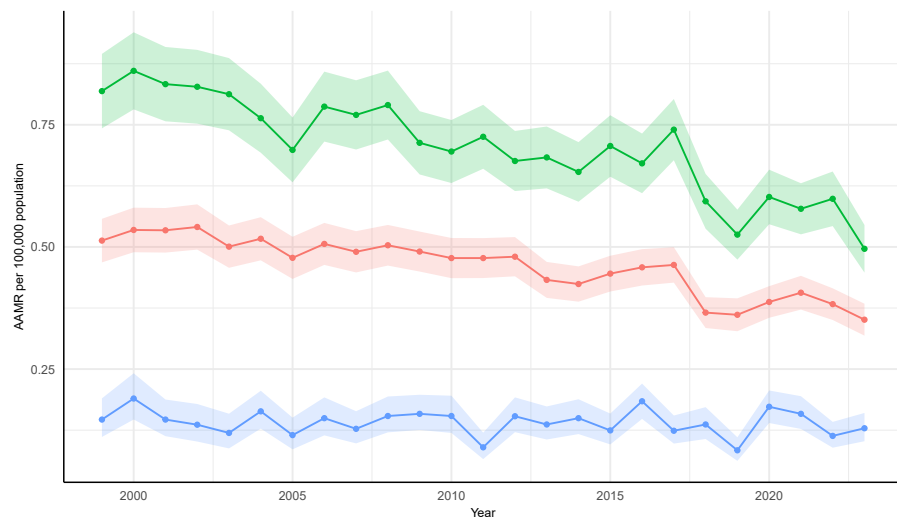

B

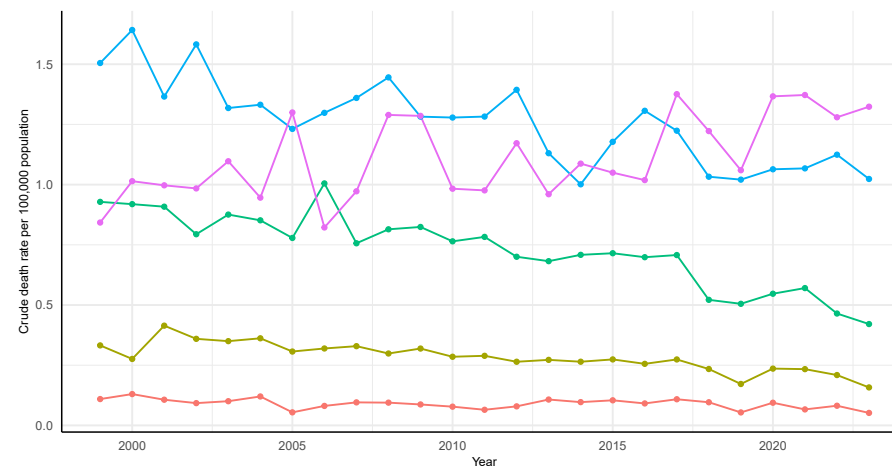

C

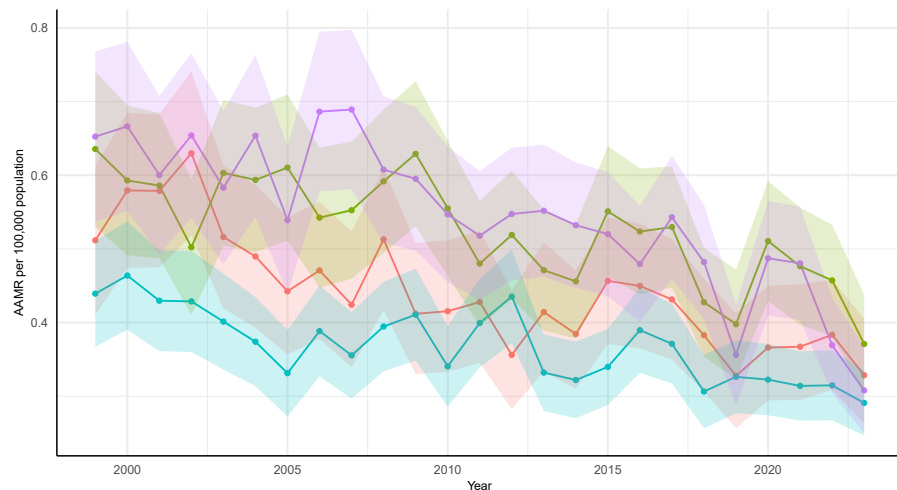

D

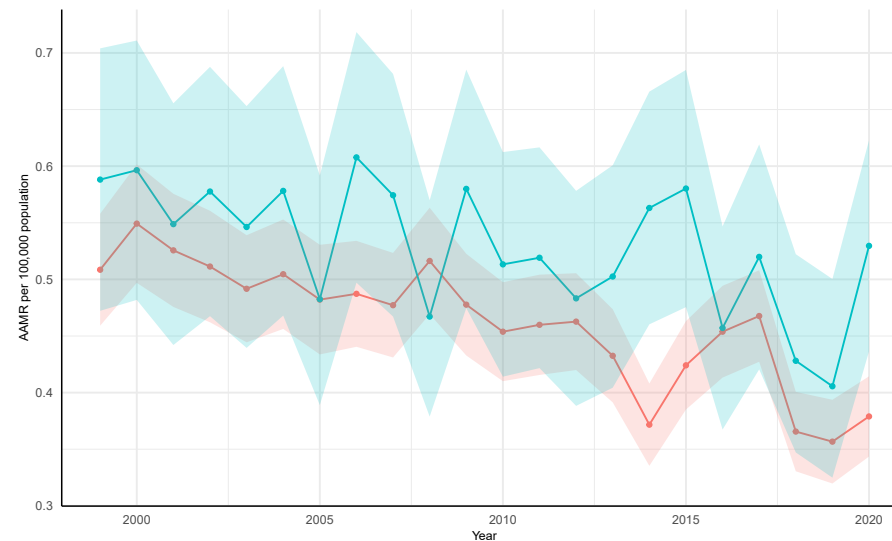

Supplement: Supplementary Figure 2 — (A) PBC AAMR by sex (1999-2023). (B) PBC crude death rate by age groups (1999-2023). (C) PBC AAMR by U.S. census regions (1999-2023). (D) PBC AAMR by urban-rural level (1999-2020). [file DataSheet2.pdf]

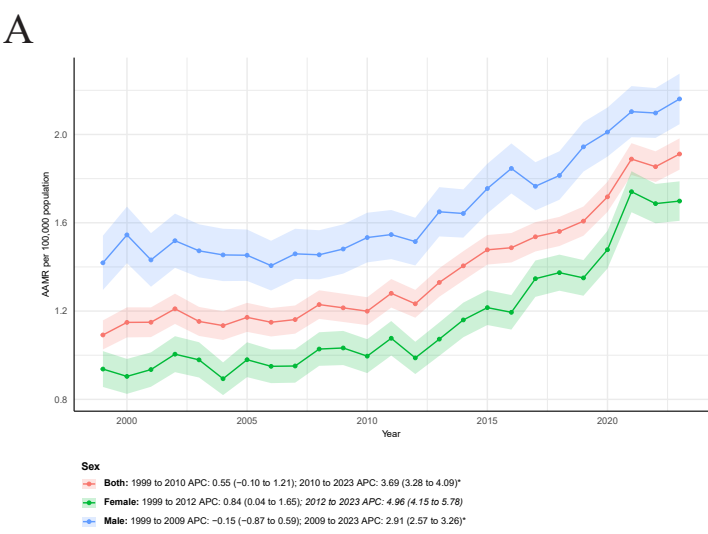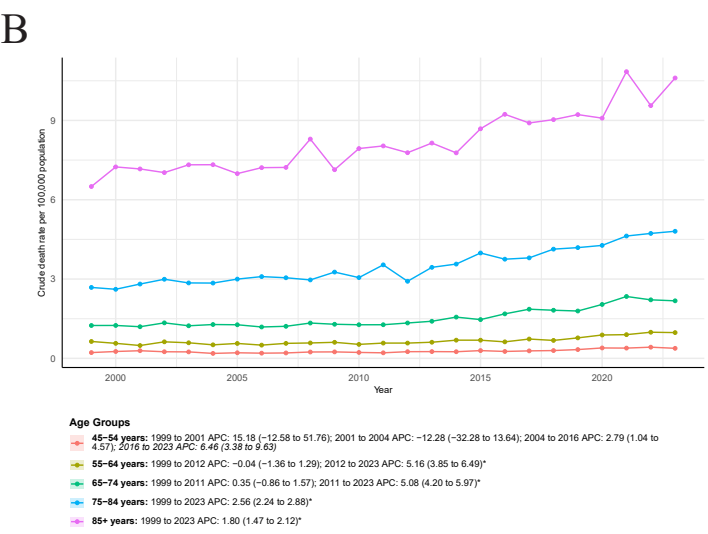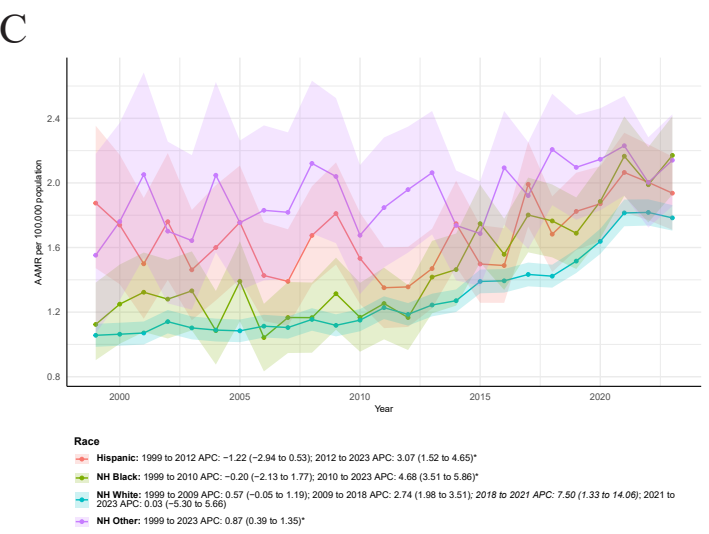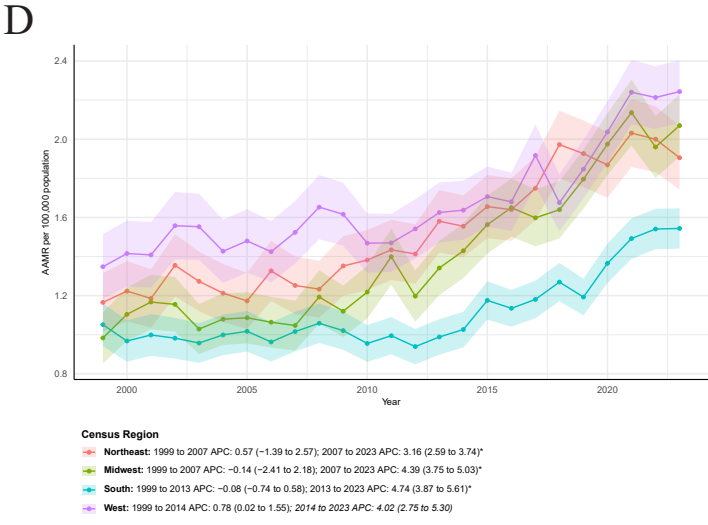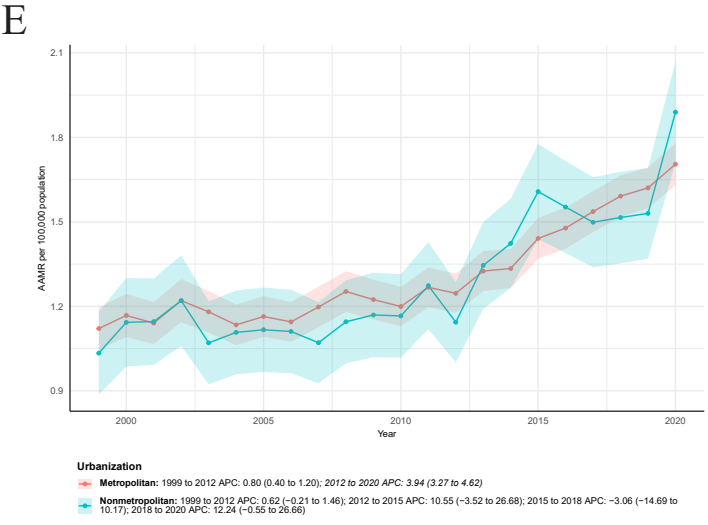

Supplement: Supplementary Figure 3 — (A) PSC AAMR by sex (1999-2023). (B) PSC crude death rate by age groups (1999-2023). (C) PSC AAMR by race/ethnicity (1999-2023). (D) PSC AAMR by U.S. census regions (1999-2023). (E) PSC AAMR by urban-rural level (1999-2020). [file DataSheet3.pdf]
