# Supplementary material for: Mortality trends and demographic-geographic disparities of autoimmune liver diseases among U.S. adults aged ≥45 years, 1999-2023
Source: Front Immunol. 2026 Feb 9;17:1762095. doi: 10.3389/fimmu.2026.1762095 (PMC12926130; doi:10.3389/fimmu.2026.1762095)
Supplement: Supplementary file 4 [file Table1.docx]

**Supplementary Tables**

Table S1 Number of deaths by sex and race/ethnicity and Corresponding population, 1999-2023.

| **Year** | **Deaths** | | | | | | | |
| --- | --- | --- | --- | --- | --- | --- | --- | --- |
|  | Overall | Male | Female | Hispanic | NH Black | NH White | NH Other | Population |
| **1999** | 1568 | 596 | 972 | 123 | 105 | 1290 | 45 | 95153686 |
| **2000** | 1624 | 647 | 977 | 117 | 124 | 1319 | 59 | 96944389 |
| **2001** | 1646 | 624 | 1022 | 106 | 129 | 1331 | 74 | 99781854 |
| **2002** | 1726 | 667 | 1059 | 139 | 129 | 1392 | 64 | 102217733 |
| **2003** | 2138 | 727 | 1411 | 170 | 174 | 1690 | 99 | 104692428 |
| **2004** | 2162 | 763 | 1399 | 193 | 154 | 1710 | 99 | 107138553 |
| **2005** | 2168 | 761 | 1407 | 193 | 188 | 1683 | 104 | 109787199 |
| **2006** | 2268 | 779 | 1489 | 189 | 169 | 1798 | 108 | 112380379 |
| **2007** | 2338 | 816 | 1522 | 208 | 192 | 1817 | 117 | 114894084 |
| **2008** | 2473 | 875 | 1598 | 246 | 177 | 1921 | 121 | 117395131 |
| **2009** | 2474 | 883 | 1591 | 243 | 201 | 1891 | 136 | 119895863 |
| **2010** | 2479 | 918 | 1561 | 227 | 191 | 1933 | 127 | 121757429 |
| **2011** | 2613 | 938 | 1675 | 243 | 197 | 2026 | 143 | 124174484 |
| **2012** | 2626 | 991 | 1635 | 262 | 211 | 2003 | 147 | 126000296 |
| **2013** | 2812 | 1063 | 1749 | 283 | 245 | 2108 | 169 | 127788037 |
| **2014** | 2924 | 1124 | 1800 | 311 | 260 | 2182 | 166 | 129779643 |
| **2015** | 3099 | 1208 | 1891 | 328 | 285 | 2311 | 163 | 131826832 |
| **2016** | 3210 | 1321 | 1889 | 310 | 310 | 2370 | 209 | 133494018 |
| **2017** | 3470 | 1311 | 2159 | 417 | 351 | 2491 | 203 | 135229289 |
| **2018** | 3393 | 1309 | 2084 | 369 | 323 | 2463 | 231 | 136335528 |
| **2019** | 3528 | 1432 | 2096 | 398 | 322 | 2591 | 213 | 137381702 |
| **2020** | 3928 | 1599 | 2329 | 450 | 400 | 2817 | 251 | 138429175 |
| **2021** | 4139 | 1624 | 2515 | 469 | 432 | 2947 | 288 | 139339453 |
| **2022** | 4195 | 1650 | 2545 | 491 | 411 | 3010 | 277 | 140311934 |
| **2023** | 4156 | 1709 | 2447 | 477 | 428 | 2936 | 306 | 141596553 |
| **Total** | 69157 | 26335 | 42822 | 6962 | 6108 | 52030 | 3919 | 3043725672 |

Table S2 Annual Percentage Change in Age-Adjusted Mortality Rates for different population subgroups across various time periods.

| **Subgroup** | **Year** | **APC (95%CI)** | **P** |
| --- | --- | --- | --- |
| Both | 1999-2004 | 5.662 (2.4668 to 8.9568) | 0.001481 |
|  | 2004-2012 | 0.5573 (-1.0425 to 2.183) | 0.47463 |
|  | 2012-2023 | 2.4645 (1.7764 to 3.1573) | 1.00E-06 |
| Female | 1999-2003 | 8.7608 (2.827 to 15.037) | 0.005364 |
|  | 2003-2023 | 1.4561 (1.0909 to 1.8227) | <0.000001 |
| Male | 1999-2023 | 2.0821 (1.8439 to 2.3209) | <0.000001 |
| 45-54 years | 1999-2023 | 2.5571 (2.0556 to 3.0611) | <0.000001 |
| 55-64 years | 1999-2023 | 1.6408 (1.15 to 2.1339) | <0.000001 |
| 65-74 years | 1999-2004 | 5.6273 (-0.0439 to 11.6203) | 0.051682 |
|  | 2004-2023 | 1.2825 (0.7264 to 1.8417) | 0.000102 |
| 75-84 years | 1999-2004 | 4.84 (1.8013 to 7.9694) | 0.003173 |
|  | 2004-2023 | 1.7547 (1.441 to 2.0693) | <0.000001 |
| 85+ years | 1999-2023 | 2.1438 (1.8311 to 2.4574) | <0.000001 |
| Hispanic | 1999-2023 | 0.8086 (0.3137 to 1.3059) | 0.002548 |
| NH Black | 1999-2023 | 2.9358 (2.4356 to 3.4384) | <0.000001 |
| NH Other | 1999-2003 | 10.132 (-1.2291 to 22.8) | 0.079292 |
|  | 2003-2023 | 0.1864 (-0.3339 to 0.7094) | 0.464247 |
| NH White | 1999-2004 | 5.2379 (2.0312 to 8.5454) | 0.002861 |
|  | 2004-2013 | 0.7469 (-0.5934 to 2.1053) | 0.257273 |
|  | 2013-2023 | 2.5631 (1.7254 to 3.4078) | 5.00E-06 |
| Midwest | 1999-2023 | 2.4788 (2.1607 to 2.7979) | <0.000001 |
| Northeast | 1999-2003 | 5.7802 (1.899 to 9.8092) | 0.006119 |
|  | 2003-2009 | 0.342 (-2.0284 to 2.7697) | 0.763899 |
|  | 2009-2021 | 2.4804 (1.8384 to 3.1265) | 1.00E-06 |
|  | 2021-2023 | -3.6352 (-11.3799 to 4.7864) | 0.359245 |
| South | 1999-2005 | 4.3286 (1.085 to 7.6764) | 0.011535 |
|  | 2005-2014 | 0.046 (-1.7057 to 1.8289) | 0.956816 |
|  | 2014-2023 | 3.5055 (2.2599 to 4.7662) | 1.40E-05 |
| West | 1999-2003 | 7.0476 (-0.4211 to 15.0763) | 0.06355 |
|  | 2003-2023 | 1.2499 (0.7906 to 1.7113) | 1.40E-05 |
| Metropolitan | 1999-2003 | 5.8588 (1.7388 to 10.1457) | 0.007621 |
|  | 2003-2020 | 1.4707 (1.1295 to 1.8131) | <0.000001 |
| Nonmetropolitan | 1999-2004 | 5.8103 (1.9868 to 9.7772) | 0.005355 |
|  | 2004-2018 | 1.2128 (0.4855 to 1.9453) | 0.002985 |
|  | 2018-2020 | 8.5325 (-3.7551 to 22.3889) | 0.165936 |

Table S3 Age-Adjusted Mortality Rates by sex (overall, male, female), 1999-2023.

| **Year** | **Age Adjusted Mortality Rate (95% CI)** | | |
| --- | --- | --- | --- |
|  | Overall | Male | Female |
| **1999** | 1.65 (1.57 to 1.73) | 1.56 (1.43 to 1.69) | 1.7 (1.59 to 1.81) |
| **2000** | 1.68 (1.6 to 1.76) | 1.71 (1.58 to 1.84) | 1.7 (1.59 to 1.81) |
| **2001** | 1.68 (1.6 to 1.76) | 1.58 (1.45 to 1.7) | 1.76 (1.65 to 1.87) |
| **2002** | 1.71 (1.63 to 1.79) | 1.63 (1.5 to 1.75) | 1.82 (1.71 to 1.93) |
| **2003** | 2.1 (2.01 to 2.19) | 1.79 (1.65 to 1.92) | 2.41 (2.28 to 2.54) |
| **2004** | 2.08 (1.99 to 2.17) | 1.8 (1.67 to 1.93) | 2.33 (2.2 to 2.45) |
| **2005** | 2.06 (1.97 to 2.14) | 1.76 (1.63 to 1.88) | 2.33 (2.21 to 2.45) |
| **2006** | 2.11 (2.03 to 2.2) | 1.75 (1.63 to 1.88) | 2.43 (2.31 to 2.56) |
| **2007** | 2.1 (2.02 to 2.19) | 1.77 (1.65 to 1.89) | 2.42 (2.3 to 2.55) |
| **2008** | 2.18 (2.09 to 2.27) | 1.89 (1.76 to 2.01) | 2.47 (2.34 to 2.59) |
| **2009** | 2.17 (2.08 to 2.25) | 1.82 (1.69 to 1.94) | 2.46 (2.33 to 2.58) |
| **2010** | 2.12 (2.04 to 2.2) | 1.88 (1.76 to 2.01) | 2.36 (2.25 to 2.48) |
| **2011** | 2.19 (2.11 to 2.28) | 1.89 (1.77 to 2.02) | 2.48 (2.36 to 2.6) |
| **2012** | 2.16 (2.08 to 2.24) | 1.89 (1.77 to 2.01) | 2.32 (2.21 to 2.43) |
| **2013** | 2.24 (2.16 to 2.33) | 2.01 (1.89 to 2.14) | 2.48 (2.37 to 2.6) |
| **2014** | 2.24 (2.16 to 2.32) | 2 (1.89 to 2.12) | 2.5 (2.38 to 2.62) |
| **2015** | 2.37 (2.28 to 2.45) | 2.15 (2.03 to 2.28) | 2.55 (2.44 to 2.67) |
| **2016** | 2.38 (2.29 to 2.46) | 2.28 (2.15 to 2.4) | 2.51 (2.4 to 2.63) |
| **2017** | 2.52 (2.43 to 2.6) | 2.19 (2.07 to 2.31) | 2.77 (2.65 to 2.89) |
| **2018** | 2.42 (2.34 to 2.51) | 2.15 (2.03 to 2.27) | 2.64 (2.53 to 2.76) |
| **2019** | 2.47 (2.39 to 2.55) | 2.3 (2.18 to 2.42) | 2.61 (2.5 to 2.73) |
| **2020** | 2.67 (2.59 to 2.76) | 2.49 (2.37 to 2.62) | 2.88 (2.76 to 3) |
| **2021** | 2.85 (2.77 to 2.94) | 2.56 (2.43 to 2.69) | 3.11 (2.98 to 3.23) |
| **2022** | 2.83 (2.75 to 2.92) | 2.51 (2.39 to 2.64) | 3.08 (2.96 to 3.2) |
| **2023** | 2.74 (2.66 to 2.83) | 2.53 (2.41 to 2.65) | 2.97 (2.85 to 3.09) |

Table S4 Age-Adjusted Mortality Rates by race/ethnicity (Hispanic, NH Black, NH White, NH Other), 1999-2023.

| **Year** | **Age Adjusted Mortality Rate (95% CI)** | | | |
| --- | --- | --- | --- | --- |
|  | Hispanic | NH Black | NH White | NH Other |
| **1999** | 2.66 (2.17 to 3.15) | 1.32 (1.06 to 1.57) | 1.62 (1.54 to 1.71) | 1.76 (1.25 to 2.4) |
| **2000** | 2.29 (1.85 to 2.72) | 1.45 (1.19 to 1.71) | 1.63 (1.54 to 1.72) | 2.2 (1.65 to 2.87) |
| **2001** | 2.11 (1.69 to 2.53) | 1.51 (1.24 to 1.77) | 1.65 (1.56 to 1.74) | 2.59 (2.01 to 3.28) |
| **2002** | 2.52 (2.08 to 2.95) | 1.5 (1.24 to 1.77) | 1.69 (1.6 to 1.78) | 2.01 (1.53 to 2.6) |
| **2003** | 2.7 (2.28 to 3.12) | 1.87 (1.59 to 2.16) | 2.02 (1.93 to 2.12) | 2.92 (2.35 to 3.58) |
| **2004** | 3.08 (2.62 to 3.53) | 1.7 (1.43 to 1.98) | 2.02 (1.92 to 2.11) | 2.91 (2.34 to 3.57) |
| **2005** | 2.9 (2.47 to 3.33) | 2.01 (1.71 to 2.3) | 1.94 (1.85 to 2.04) | 2.77 (2.22 to 3.32) |
| **2006** | 2.65 (2.26 to 3.05) | 1.66 (1.41 to 1.92) | 2.05 (1.96 to 2.15) | 2.85 (2.29 to 3.41) |
| **2007** | 2.73 (2.34 to 3.12) | 1.88 (1.61 to 2.15) | 2.07 (1.98 to 2.17) | 2.81 (2.28 to 3.34) |
| **2008** | 3.15 (2.74 to 3.56) | 1.71 (1.45 to 1.97) | 2.12 (2.02 to 2.21) | 2.89 (2.36 to 3.42) |
| **2009** | 2.96 (2.57 to 3.35) | 1.87 (1.6 to 2.14) | 2.08 (1.99 to 2.18) | 2.98 (2.46 to 3.5) |
| **2010** | 2.6 (2.24 to 2.95) | 1.76 (1.5 to 2.01) | 2.1 (2 to 2.19) | 2.7 (2.21 to 3.18) |
| **2011** | 2.53 (2.2 to 2.87) | 1.81 (1.55 to 2.07) | 2.15 (2.06 to 2.25) | 2.8 (2.33 to 3.28) |
| **2012** | 2.62 (2.29 to 2.95) | 1.73 (1.49 to 1.98) | 2.05 (1.96 to 2.14) | 2.77 (2.31 to 3.23) |
| **2013** | 2.75 (2.41 to 3.08) | 2.08 (1.81 to 2.35) | 2.18 (2.08 to 2.27) | 2.88 (2.44 to 3.33) |
| **2014** | 2.87 (2.54 to 3.2) | 2.07 (1.81 to 2.33) | 2.2 (2.1 to 2.29) | 2.63 (2.22 to 3.04) |
| **2015** | 2.87 (2.55 to 3.19) | 2.26 (1.99 to 2.53) | 2.3 (2.2 to 2.39) | 2.5 (2.11 to 2.89) |
| **2016** | 2.58 (2.28 to 2.88) | 2.32 (2.05 to 2.59) | 2.29 (2.2 to 2.38) | 3.02 (2.6 to 3.44) |
| **2017** | 3.39 (3.06 to 3.73) | 2.52 (2.25 to 2.79) | 2.4 (2.3 to 2.49) | 2.86 (2.46 to 3.26) |
| **2018** | 2.74 (2.45 to 3.03) | 2.32 (2.06 to 2.58) | 2.33 (2.24 to 2.43) | 3.06 (2.65 to 3.46) |
| **2019** | 2.96 (2.66 to 3.26) | 2.22 (1.97 to 2.47) | 2.38 (2.28 to 2.47) | 2.68 (2.32 to 3.05) |
| **2020** | 3.01 (2.73 to 3.3) | 2.73 (2.46 to 3.01) | 2.59 (2.5 to 2.69) | 2.95 (2.58 to 3.33) |
| **2021** | 3.16 (2.86 to 3.46) | 2.97 (2.68 to 3.26) | 2.78 (2.67 to 2.88) | 3.09 (2.73 to 3.45) |
| **2022** | 3.2 (2.91 to 3.49) | 2.77 (2.49 to 3.04) | 2.77 (2.67 to 2.87) | 2.72 (2.4 to 3.05) |
| **2023** | 2.99 (2.71 to 3.26) | 2.84 (2.56 to 3.12) | 2.66 (2.56 to 2.75) | 2.91 (2.58 to 3.24) |

Table S5 Age-Adjusted Mortality Rates by U.S. Census Region (Northeast, Midwest, South, West), 1999-2023.

| **Year** | **Age Adjusted Mortality Rate (95% CI)** | | | |
| --- | --- | --- | --- | --- |
|  | Northeast | Midwest | South | West |
| **1999** | 1.66 (1.48 to 1.84) | 1.62 (1.45 to 1.79) | 1.5 (1.37 to 1.64) | 1.98 (1.77 to 2.18) |
| **2000** | 1.8 (1.61 to 1.98) | 1.7 (1.53 to 1.87) | 1.41 (1.29 to 1.54) | 2.09 (1.88 to 2.29) |
| **2001** | 1.77 (1.59 to 1.95) | 1.77 (1.59 to 1.94) | 1.43 (1.3 to 1.56) | 1.97 (1.77 to 2.17) |
| **2002** | 1.95 (1.76 to 2.14) | 1.62 (1.46 to 1.78) | 1.41 (1.29 to 1.54) | 2.21 (2.01 to 2.42) |
| **2003** | 2.1 (1.91 to 2.3) | 2.09 (1.9 to 2.27) | 1.73 (1.6 to 1.87) | 2.68 (2.46 to 2.9) |
| **2004** | 2.07 (1.88 to 2.27) | 2.13 (1.94 to 2.31) | 1.8 (1.67 to 1.94) | 2.57 (2.35 to 2.78) |
| **2005** | 2.02 (1.83 to 2.21) | 2.14 (1.95 to 2.32) | 1.73 (1.6 to 1.87) | 2.53 (2.32 to 2.75) |
| **2006** | 2.18 (1.98 to 2.38) | 2.07 (1.89 to 2.25) | 1.75 (1.62 to 1.88) | 2.78 (2.56 to 3) |
| **2007** | 2.14 (1.94 to 2.33) | 2.09 (1.91 to 2.27) | 1.79 (1.66 to 1.92) | 2.7 (2.49 to 2.92) |
| **2008** | 2.09 (1.9 to 2.28) | 2.15 (1.97 to 2.33) | 1.92 (1.78 to 2.05) | 2.82 (2.61 to 3.04) |
| **2009** | 2.11 (1.92 to 2.3) | 2.16 (1.98 to 2.34) | 1.77 (1.64 to 1.9) | 2.8 (2.59 to 3.01) |
| **2010** | 2.2 (2 to 2.39) | 2.24 (2.05 to 2.42) | 1.77 (1.64 to 1.89) | 2.54 (2.34 to 2.73) |
| **2011** | 2.27 (2.08 to 2.47) | 2.29 (2.11 to 2.47) | 1.83 (1.7 to 1.96) | 2.58 (2.38 to 2.78) |
| **2012** | 2.12 (1.94 to 2.31) | 2.2 (2.02 to 2.38) | 1.77 (1.65 to 1.9) | 2.67 (2.48 to 2.87) |
| **2013** | 2.36 (2.16 to 2.55) | 2.32 (2.13 to 2.5) | 1.78 (1.66 to 1.9) | 2.76 (2.56 to 2.96) |
| **2014** | 2.35 (2.15 to 2.54) | 2.52 (2.33 to 2.7) | 1.76 (1.64 to 1.88) | 2.7 (2.51 to 2.89) |
| **2015** | 2.5 (2.3 to 2.7) | 2.53 (2.34 to 2.71) | 1.88 (1.76 to 2.01) | 2.84 (2.64 to 3.04) |
| **2016** | 2.48 (2.28 to 2.68) | 2.68 (2.49 to 2.88) | 1.95 (1.83 to 2.07) | 2.74 (2.55 to 2.94) |
| **2017** | 2.54 (2.35 to 2.74) | 2.65 (2.46 to 2.83) | 2.11 (1.98 to 2.23) | 3.05 (2.85 to 3.25) |
| **2018** | 2.78 (2.57 to 2.98) | 2.61 (2.43 to 2.8) | 1.93 (1.81 to 2.05) | 2.74 (2.55 to 2.92) |
| **2019** | 2.7 (2.5 to 2.9) | 2.63 (2.44 to 2.81) | 2 (1.88 to 2.12) | 2.83 (2.64 to 3.01) |
| **2020** | 2.7 (2.5 to 2.9) | 3.07 (2.87 to 3.27) | 2.29 (2.16 to 2.42) | 3.07 (2.88 to 3.26) |
| **2021** | 2.82 (2.61 to 3.02) | 3.15 (2.95 to 3.35) | 2.39 (2.26 to 2.52) | 3.43 (3.23 to 3.63) |
| **2022** | 2.79 (2.59 to 2.99) | 2.98 (2.79 to 3.18) | 2.43 (2.3 to 2.56) | 3.33 (3.13 to 3.53) |
| **2023** | 2.61 (2.42 to 2.8) | 2.97 (2.78 to 3.16) | 2.35 (2.23 to 2.48) | 3.24 (3.05 to 3.43) |

Table S6 Age-Adjusted Mortality Rates by urbanization level (Metropolitan, Nonmetropolitan), 1999-2020.

| **Year** | **Age Adjusted Mortality Rate (95% CI)** | |
| --- | --- | --- |
|  | Metropolitan | Nonmetropolitan |
| **1999** | 1.63 (1.54 to 1.72) | 1.61 (1.43 to 1.8) |
| **2000** | 1.67 (1.58 to 1.76) | 1.74 (1.55 to 1.93) |
| **2001** | 1.67 (1.58 to 1.76) | 1.74 (1.55 to 1.93) |
| **2002** | 1.7 (1.61 to 1.79) | 1.8 (1.6 to 1.99) |
| **2003** | 2.12 (2.02 to 2.22) | 2.09 (1.89 to 2.3) |
| **2004** | 2.06 (1.97 to 2.16) | 2.23 (2.02 to 2.45) |
| **2005** | 2.04 (1.94 to 2.13) | 2.1 (1.89 to 2.3) |
| **2006** | 2.09 (1.99 to 2.19) | 2.2 (2 to 2.41) |
| **2007** | 2.08 (1.99 to 2.18) | 2.15 (1.95 to 2.35) |
| **2008** | 2.2 (2.1 to 2.29) | 2.09 (1.89 to 2.29) |
| **2009** | 2.13 (2.03 to 2.22) | 2.25 (2.04 to 2.45) |
| **2010** | 2.11 (2.02 to 2.2) | 2.24 (2.03 to 2.45) |
| **2011** | 2.15 (2.06 to 2.25) | 2.37 (2.16 to 2.59) |
| **2012** | 2.16 (2.06 to 2.25) | 2.14 (1.94 to 2.34) |
| **2013** | 2.18 (2.09 to 2.27) | 2.38 (2.18 to 2.59) |
| **2014** | 2.2 (2.11 to 2.28) | 2.57 (2.35 to 2.78) |
| **2015** | 2.29 (2.2 to 2.38) | 2.64 (2.43 to 2.86) |
| **2016** | 2.38 (2.29 to 2.47) | 2.51 (2.3 to 2.72) |
| **2017** | 2.56 (2.46 to 2.65) | 2.43 (2.23 to 2.63) |
| **2018** | 2.4 (2.31 to 2.49) | 2.47 (2.26 to 2.67) |
| **2019** | 2.46 (2.37 to 2.55) | 2.45 (2.25 to 2.65) |
| **2020** | 2.63 (2.54 to 2.72) | 3.09 (2.86 to 3.31) |

Table S7 Age-adjusted mortality rates (AAMR) and annual percent changes (APC/AAPC) for AIH in the United States, 1999-2023.

| Characteristics | Deaths (2003) | Deaths (2023) | Percent Change (%) | AAMR (2003) | AAMR (2023) | AAPC (95% CI) |
| --- | --- | --- | --- | --- | --- | --- |
| **Overall** | 435 | 841 | 93.33 | 0.42 (0.38 to 0.46) | 0.56 (0.52 to 0.60) | 1.77 (1.19 to 2.35) * |
| **Sex** |  |  |  |  |  |  |
| Female | 362 | 648 | 79.01 | 0.65 (0.58 to 0.72) | 0.78 (0.72 to 0.84) | 1.25 (0.27 to 2.24) * |
| Male | 73 | 193 | 164.38 | 0.16 (0.12 to 0.20) | 0.29 (0.25 to 0.33) | 3.14 (2.32 to 3.96) * |
| **Census Region** |  |  |  |  |  |  |
| Northeast | 62 | 104 | 67.74 | 0.32 (0.24 to 0.41) | 0.39 (0.31 to 0.46) | 1.38 (0.52 to 2.26) * |
| Midwest | 101 | 182 | 80.20 | 0.39 (0.32 to 0.47) | 0.59 (0.50 to 0.67) | 1.55 (0.81 to 2.30) * |
| South | 149 | 315 | 111.41 | 0.41 (0.34 to 0.47) | 0.52 (0.47 to 0.58) | 1.87 (0.98 to 2.78) * |
| West | 123 | 240 | 95.12 | 0.61 (0.50 to 0.71) | 0.70 (0.62 to 0.79) | 1.39 (0.76 to 2.02) * |
| **Race** |  |  |  |  |  |  |
| Hispanic | 45 | 123 | 173.33 | 0.62 (0.45 to 0.84) | 0.74 (0.60 to 0.87) | 0.92 (0.20 to 1.65) * |
| NH Black | 41 | 88 | 114.63 | 0.39 (0.28 to 0.54) | 0.58 (0.46 to 0.72) | 2.37 (1.07 to 3.68) * |
| NH White | 324 | 564 | 74.07 | 0.38 (0.34 to 0.42) | 0.52 (0.47 to 0.56) | 1.65 (1.12 to 2.18) * |
| **Urbanization##** |  |  |  |  |  |  |
| Metropolitan | 344 | 688 | 100 | 0.38 (0.34 to 0.43) | 0.57 (0.53 to 0.61) | 1.71 (1.04 to 2.38) * |
| Nonmetropolitan | 91 | 153 | 68.13 | 0.49 (0.39 to 0.60) | 0.76 (0.65 to 0.88) | 1.86 (-0.59 to 4.37) |
| **Age#** |  |  |  |  |  |  |
| 45-54 years | 53 | 81 | 52.83 | 0.13 (0.10 to 0.17) | 0.20 (0.16 to 0.25) | 2.68 (1.62 to 3.74) * |
| 55-64 years | 100 | 157 | 57.00 | 0.36 (0.29 to 0.43) | 0.38 (0.32 to 0.43) | 0.97 (0.17 to 1.77) * |
| 65-74 years | 136 | 258 | 89.71 | 0.74 (0.61 to 0.86) | 0.74 (0.65 to 0.83) | 0.27 (-0.22 to 0.76) |
| 75-84 years | 125 | 248 | 98.40 | 0.97 (0.80 to 1.14) | 1.35 (1.18 to 1.52) | 1.89 (1.17 to 2.60) * |
| 85+ years | 21 | 97 | 361.90 | 0.47 (0.29 to 0.72) | 1.57 (1.27 to 1.91) | 4.27 (2.72 to 5.84) * |

The AAPC was calculated for the period 1999–2023. # Age groups used crude mortality rates for calculation. ## Urban-rural analysis (AAMR and AAPC) is for the period 1999-2020. The table lists the death numbers for 2023, but due to the lack of urban-rural classification data after 2020, the corresponding 2023 AAMR has not been calculated. The AAMR values shown for urbanization are for the year 2020. * Means statistical significance.

Table S8 Age-adjusted mortality rates (AAMR) and annual percent changes (APC/AAPC) for PBC in the United States, 1999-2023.

| Characteristics | Deaths (1999) | Deaths (2023) | Percent Change (%) | AAMR (1999) | AAMR (2023) | AAPC (95% CI) |
| --- | --- | --- | --- | --- | --- | --- |
| **Overall** | 509 | 503 | -1.18 | 0.51 (0.47 to 0.56) | 0.35 (0.32 to 0.38) | -1.56 (-1.88 to -1.23) * |
| **Sex** |  |  |  |  |  |  |
| Female | 446 | 411 | -7.85 | 0.82 (0.74 to 0.90) | 0.50 (0.45 to 0.54) | -1.74 (-2.12 to -1.36) * |
| Male | 63 | 92 | 46.03 | 0.15 (0.11 to 0.19) | 0.13 (0.10 to 0.16) | -0.43 (-1.49 to 0.65) |
| **Census** **Region** |  |  |  |  |  |  |
| Northeast | 102 | 90 | -11.76 | 0.51 (0.41 to 0.61) | 0.33 (0.26 to 0.40) | -1.98 (-2.51 to -1.44) * |
| Midwest | 140 | 124 | -11.43 | 0.64 (0.53 to 0.74) | 0.37 (0.30 to 0.44) | -1.44 (-1.96 to -0.92) * |
| South | 144 | 176 | 22.22 | 0.44 (0.37 to 0.51) | 0.29 (0.25 to 0.34) | -1.40 (-1.88 to -0.92) * |
| West | 123 | 113 | -8.13 | 0.65 (0.54 to 0.77) | 0.31 (0.25 to 0.37) | -3.31 (-5.57 to -1.00) * |
| **Race** |  |  |  |  |  |  |
| Hispanic | 43 | 60 | 39.53 | 0.78 (0.56 to 1.06) | 0.36 (0.27 to 0.47) | -2.36 (-3.22 to -1.49) * |
| NH White | 445 | 395 | -11.24 | 0.57 (0.52 to 0.63) | 0.33 (0.30 to 0.37) | -1.62 (-2.01 to -1.24) * |
| **Urbanization##** |  |  |  |  |  |  |
| Metropolitan | 408 | 407 | -0.25 | 0.51 (0.46 to 0.56) | 0.38 (0.34 to 0.41) | -1.55 (-2.01 to -1.09) * |
| Nonmetropolitan | 101 | 96 | -4.95 | 0.59 (0.47 to 0.70) | 0.53 (0.44 to 0.62) | -0.96 (-1.59 to -0.32) * |
| **Age#** |  |  |  |  |  |  |
| 45-54 years | 40 | 21 | -47.50 | 0.11 (0.08 to 0.15) | 0.05 (0.03 to 0.08) | -1.34 (-2.51 to -0.15) * |
| 55-64 years | 79 | 66 | -16.46 | 0.33 (0.26 to 0.41) | 0.16 (0.12 to 0.20) | -2.52 (-3.15 to -1.89) * |
| 65-74 years | 171 | 146 | -14.62 | 0.93 (0.79 to 1.07) | 0.42 (0.35 to 0.49) | -3.09 (-4.12 to -2.04) * |
| 75-84 years | 184 | 188 | 2.17 | 1.51 (1.29 to 1.72) | 1.02 (0.88 to 1.17) | -1.57 (-2.00 to -1.13) * |
| 85+ years | 35 | 82 | 134.29 | 0.84 (0.59 to 1.17) | 1.32 (1.05 to 1.64) | 1.26 (0.52 to 2.00) * |

The AAPC was calculated for the period 1999–2023. # Age groups used crude mortality rates for calculation. ## Urban-rural analysis (AAMR and AAPC) is for the period 1999-2020. The table lists the death numbers for 2023, but due to the lack of urban-rural classification data after 2020, the corresponding 2023 AAMR has not been calculated. The AAMR values shown for urbanization are for the year 2020. * Means statistical significance.

Table S9 Age-adjusted mortality rates (AAMR) and annual percent changes (APC/AAPC) for PSC in the United States, 1999-2023.

| Characteristics | Deaths (1999) | Deaths (2023) | Percent Change (%) | AAMR (1999) | AAMR (2023) | AAPC (95% CI) |
| --- | --- | --- | --- | --- | --- | --- |
| **Overall** | 1059 | 2857 | 169.78 | 1.09 (1.03 to 1.16) | 1.91 (1.84 to 1.98) | 2.24 (1.89 to 2.59) * |
| **Sex** |  |  |  |  |  |  |
| Female | 526 | 1425 | 170.91 | 0.94 (0.86 to 1.02) | 1.70 (1.61 to 1.79) | 2.71 (2.17 to 3.25) * |
| Male | 533 | 1432 | 168.67 | 1.42 (1.30 to 1.54) | 2.16 (2.05 to 2.28) | 1.63 (1.28 to 1.97) * |
| **Census Region** |  |  |  |  |  |  |
| Northeast | 234 | 539 | 130.34 | 1.17 (1.02 to 1.32) | 1.91 (1.74 to 2.07) | 2.29 (1.57 to 3.02) * |
| Midwest | 221 | 666 | 201.36 | 0.98 (0.85 to 1.11) | 2.07 (1.91 to 2.23) | 2.86 (2.02 to 3.70) * |
| South | 353 | 886 | 150.99 | 1.05 (0.94 to 1.16) | 1.54 (1.44 to 1.65) | 1.90 (1.40 to 2.40) * |
| West | 251 | 766 | 205.18 | 1.35 (1.18 to 1.51) | 2.24 (2.08 to 2.40) | 1.98 (1.35 to 2.62) * |
| **Race** |  |  |  |  |  |  |
| Hispanic | 80 | 299 | 273.75 | 1.88 (1.47 to 2.35) | 1.94 (1.71 to 2.16) | 0.72 (-0.39 to 1.85) |
| NH Black | 92 | 325 | 253.26 | 1.12 (0.90 to 1.38) | 2.17 (1.93 to 2.41) | 2.41 (1.38 to 3.46) * |
| NH White | 845 | 2006 | 137.40 | 1.06 (0.99 to 1.13) | 1.78 (1.70 to 1.86) | 2.18 (1.30 to 3.07) * |
| NH Other | 37 | 223 | 502.70 | 1.55 (1.07 to 2.18) | 2.14 (1.86 to 2.42) | 0.87 (0.39 to 1.35) * |
| **Urbanization##** |  |  |  |  |  |  |
| Metropolitan | 869 | 2387 | 174.68 | 1.12 (1.05 to 1.20) | 1.70 (1.63 to 1.78) | 1.99 (1.66 to 2.32) * |
| Nonmetropolitan | 190 | 470 | 147.37 | 1.03 (0.89 to 1.18) | 1.89 (1.71 to 2.07) | 2.50 (-0.16 to 5.23) |
| **Age#** |  |  |  |  |  |  |
| 45-54 years | 80 | 154 | 92.50 | 0.22 (0.17 to 0.27) | 0.38 (0.32 to 0.44) | 2.78 (-1.05 to 6.75) |
| 55-64 years | 152 | 408 | 168.42 | 0.64 (0.54 to 0.74) | 0.97 (0.88 to 1.07) | 2.31 (1.43 to 3.20) * |
| 65-74 years | 229 | 755 | 229.69 | 1.24 (1.08 to 1.40) | 2.18 (2.02 to 2.33) | 2.69 (1.98 to 3.40) * |
| 75-84 years | 328 | 883 | 169.21 | 2.68 (2.39 to 2.97) | 4.81 (4.49 to 5.12) | 2.56 (2.24 to 2.88) * |
| 85+ years | 270 | 657 | 143.33 | 6.50 (5.72 to 7.28) | 10.61 (9.79 to 11.42) | 1.80 (1.47 to 2.12) * |

The AAPC was calculated for the period 1999–2023. # Age groups used crude mortality rates for calculation. ## Urban-rural analysis (AAMR and AAPC) is for the period 1999-2020. The table lists the death numbers for 2023, but due to the lack of urban-rural classification data after 2020, the corresponding 2023 AAMR has not been calculated. The AAMR values shown for urbanization are for the year 2020. * Means statistical significance.
